# Supplementary material for: Room-temperature memristive switching between charge density wave states
Source: Nat Commun. 2026 May 21;17:8360. doi: 10.1038/s41467-026-73267-x (PMC13473091; doi:10.1038/s41467-026-73267-x)
Supplement: Supplementary file 1 [file 41467_2026_73267_MOESM1_ESM.pdf]

Supplementary Information

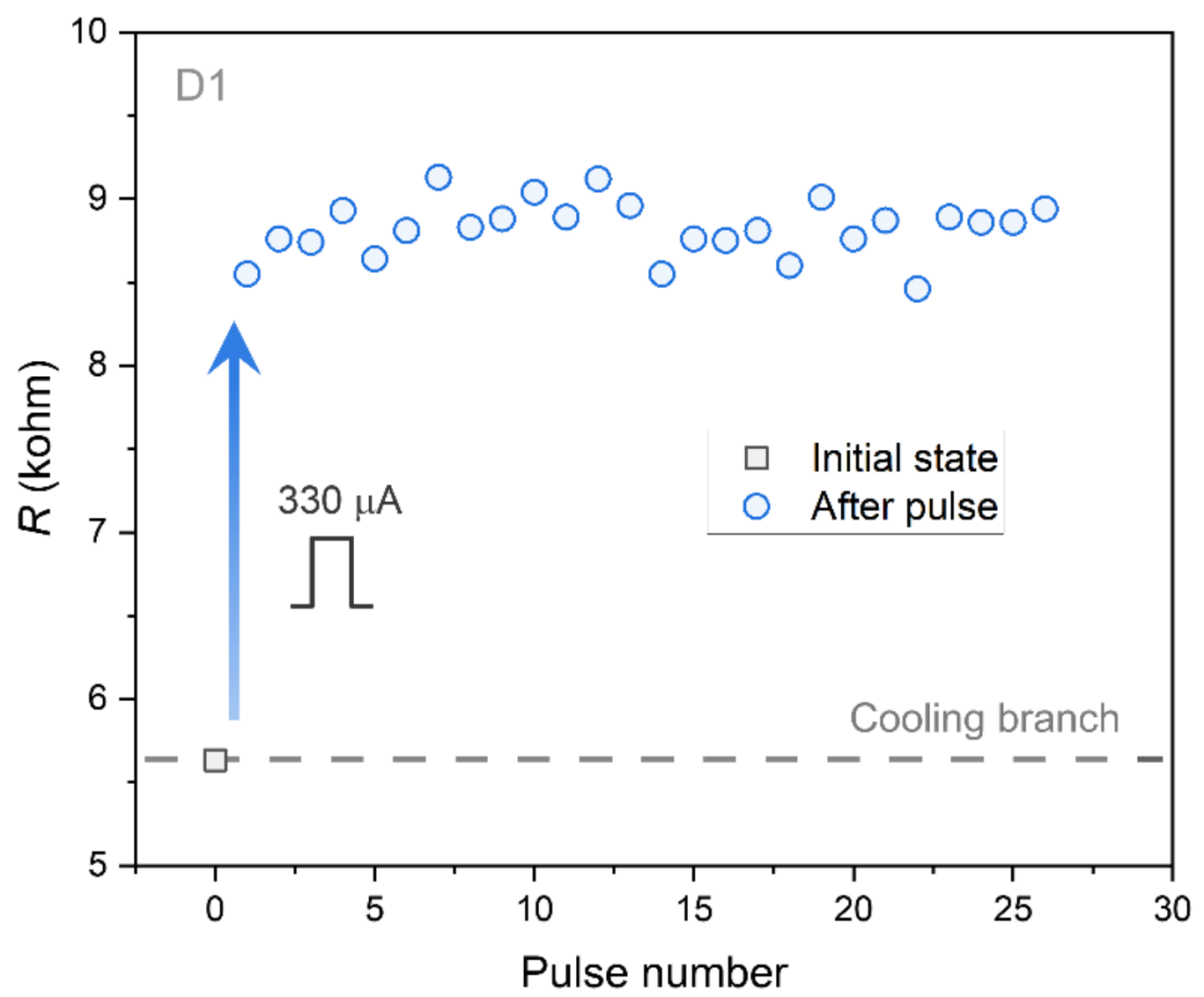

Supplementary Fig. 1. A sequence of 330 μA current pulses is applied at 300 K to the device D1, starting from the cooling branch.

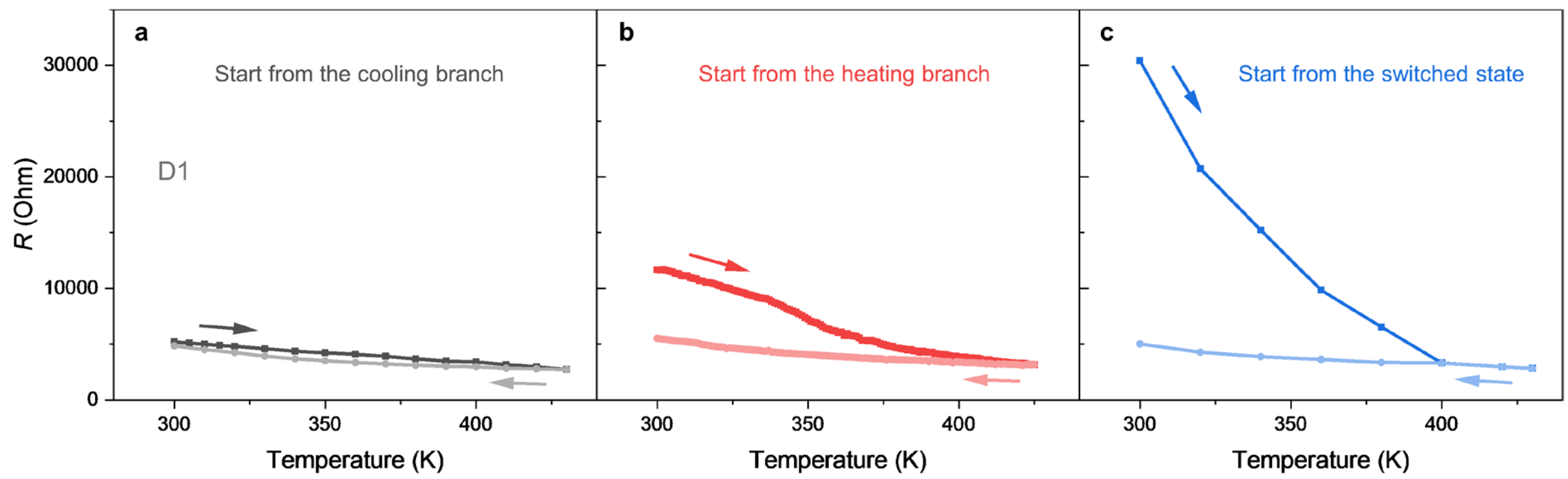

Supplementary Fig. 2. a-c, Thermal cycle starting from the cooling branch (**a**), the heating branch (**b**), and the switched state (**c**).

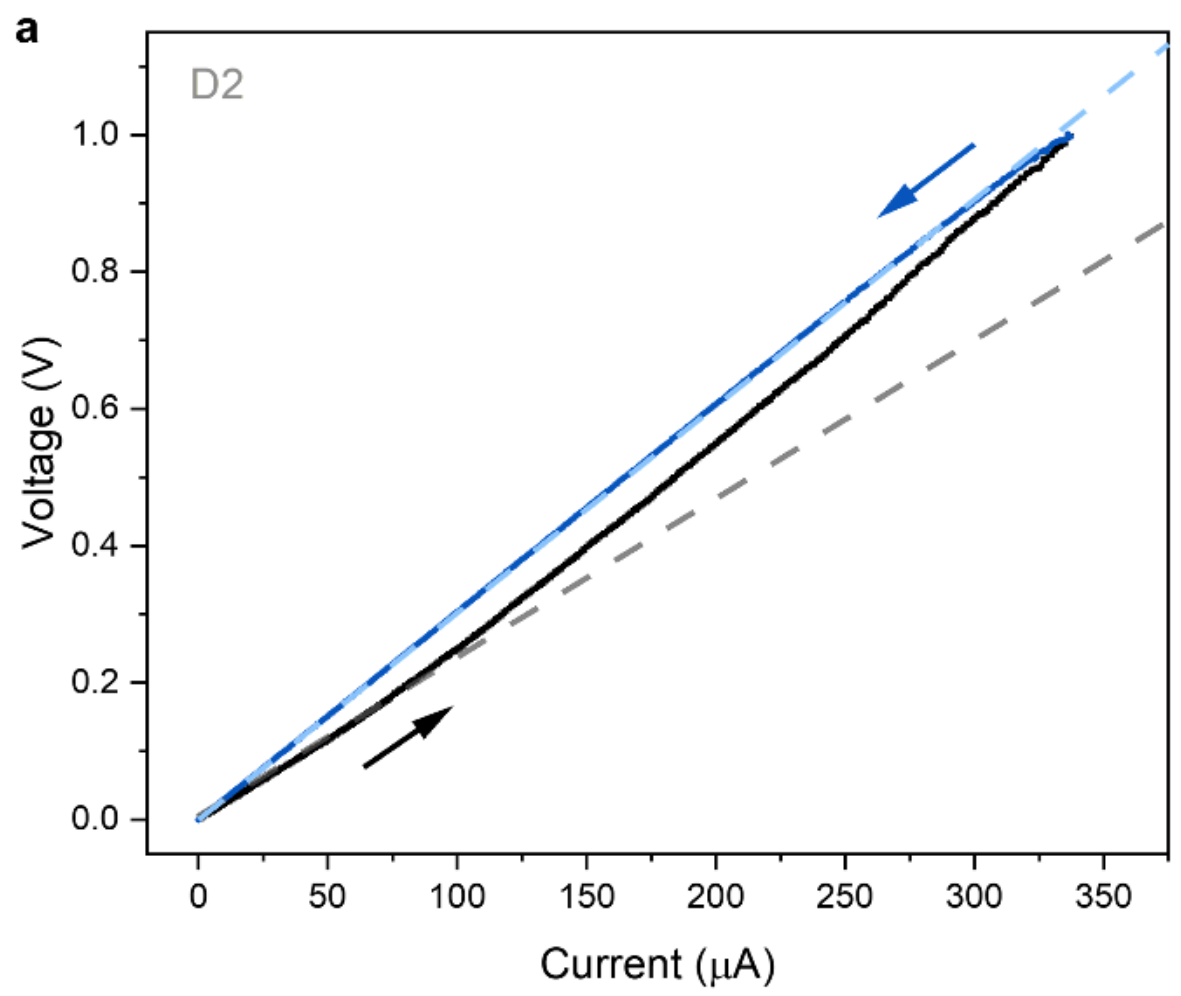

Supplementary Fig. 3. a, Voltage measured during the current pulses by incrementally increasing (black) and decreasing (blue) pulse magnitude. Dashed lines are a fit to measurements up to 0.1 V. Deviation from the linear fit is due to resistance switching. **b**, Same as (**a**) with an additional measurement of increasing pulse magnitude (green). The additional measurement of incrementally increasing current in an already switched state follows linear behavior.

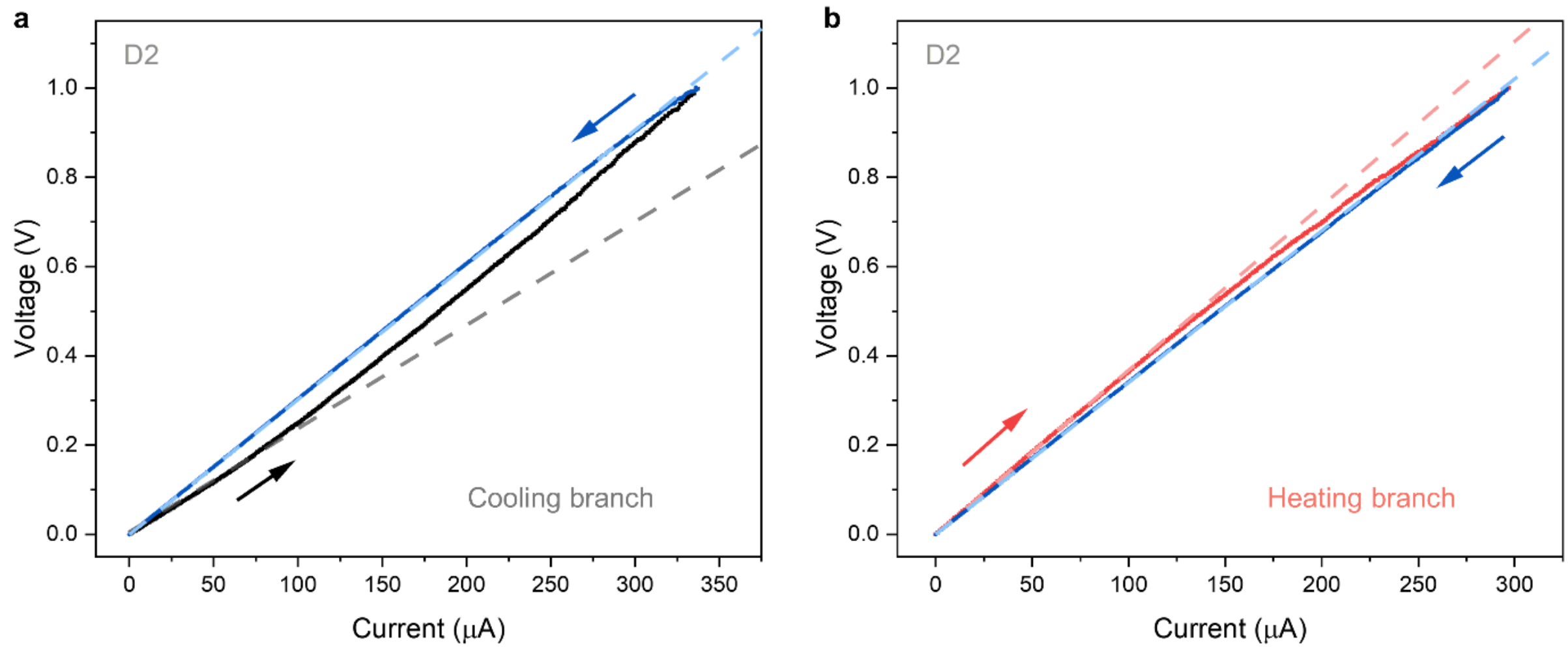

Supplementary Fig. 4. a, Voltage measured during the current pulses by incrementally increasing (black) and decreasing (blue) pulse magnitude starting from the cooling branch. **b**, Voltage measured during the current pulses by incrementally increasing (red) and decreasing (blue) pulse magnitude starting from the heating branch. All measurements were done at 300 K. Dashed lines indicate a linear fit to the curve in the interval between 0 and 0.1 V.

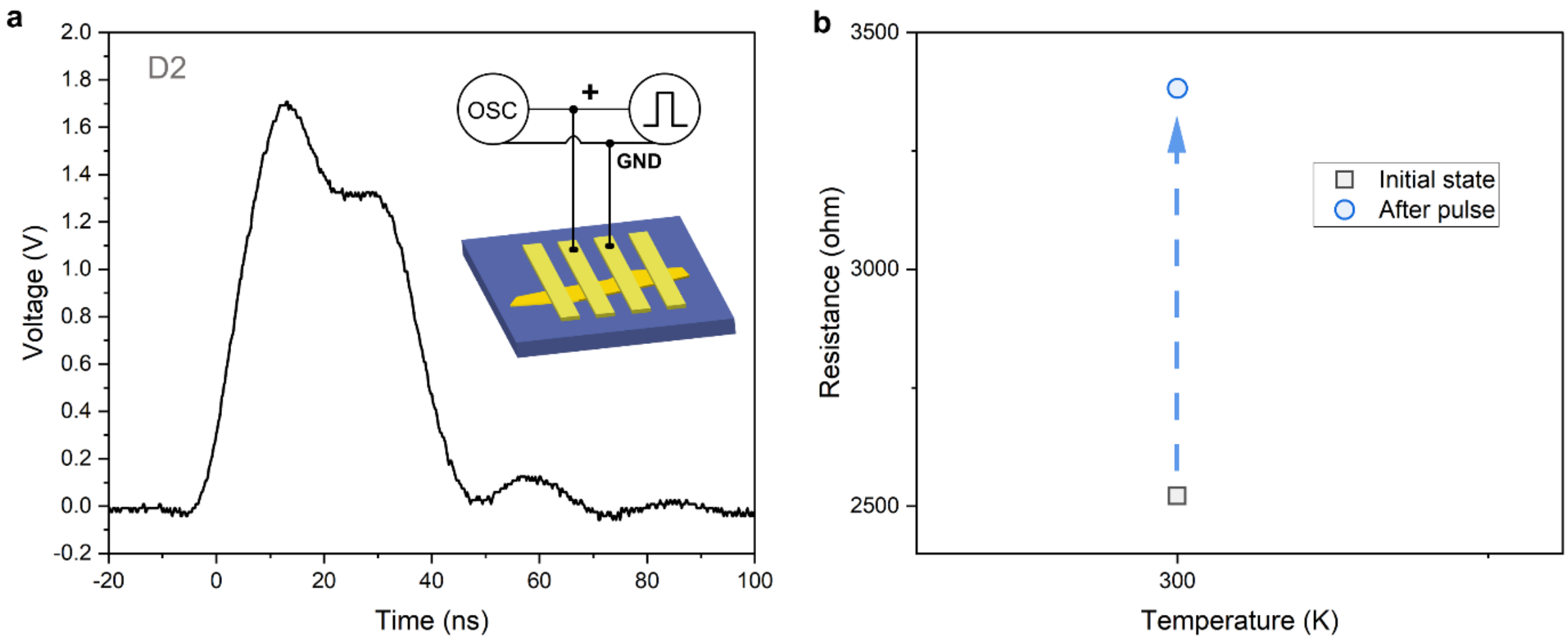

Supplementary Fig. 5. a, Voltage over the device during the 30 ns voltage pulse. The inset figure shows a schematic of the device connection to an oscilloscope and a voltage pulse source. **b**, Change in the device resistance after the 30 ns pulse (shown in Supplementary Fig. 5a) is applied.

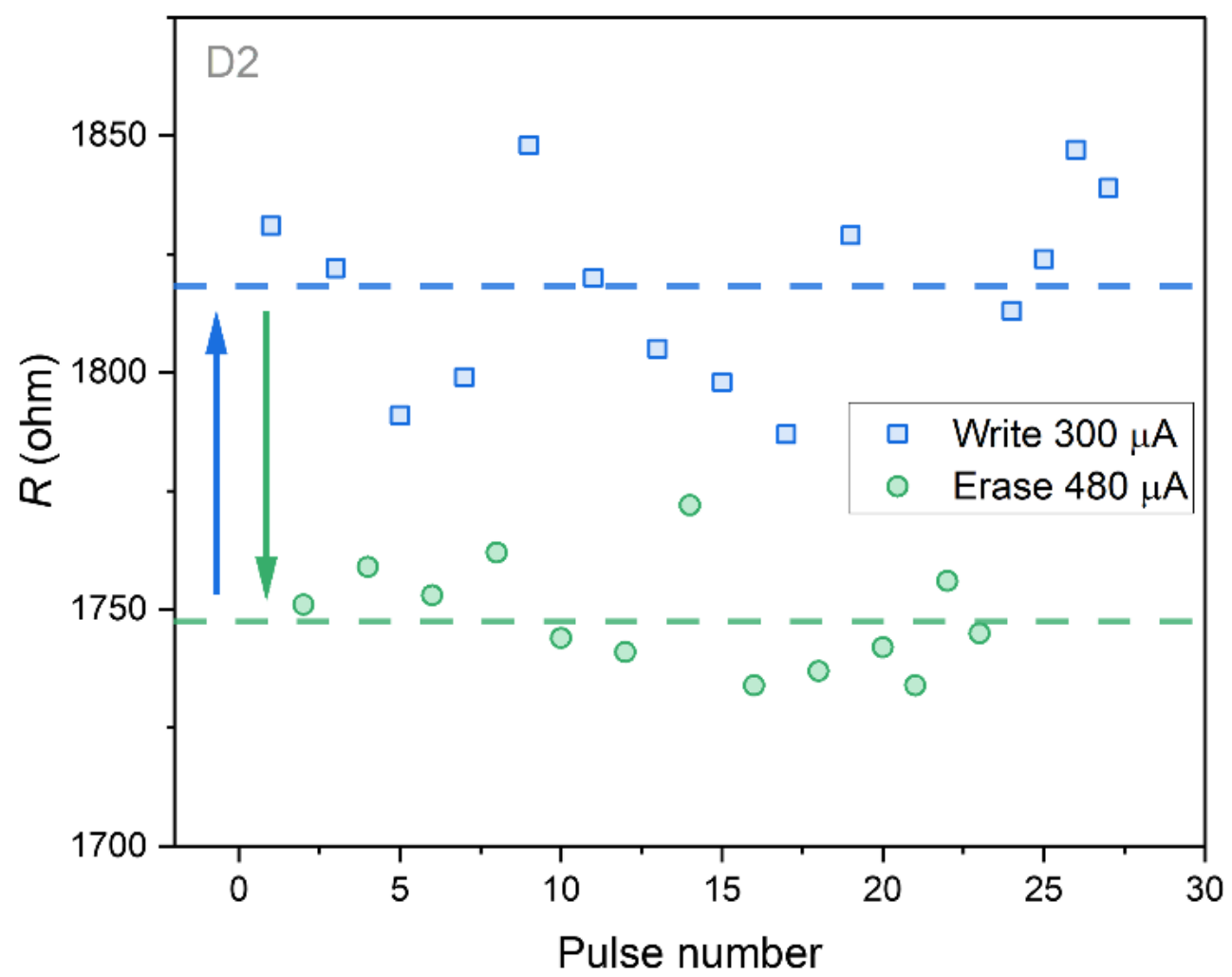

Supplementary Fig. 6. By applying either moderate (write) or high-current (erase) pulses, the resistance can be controllably switched via electrical pulses in both directions. We attribute the drop in resistance after the high-current pulses to the Joule heating of the device.

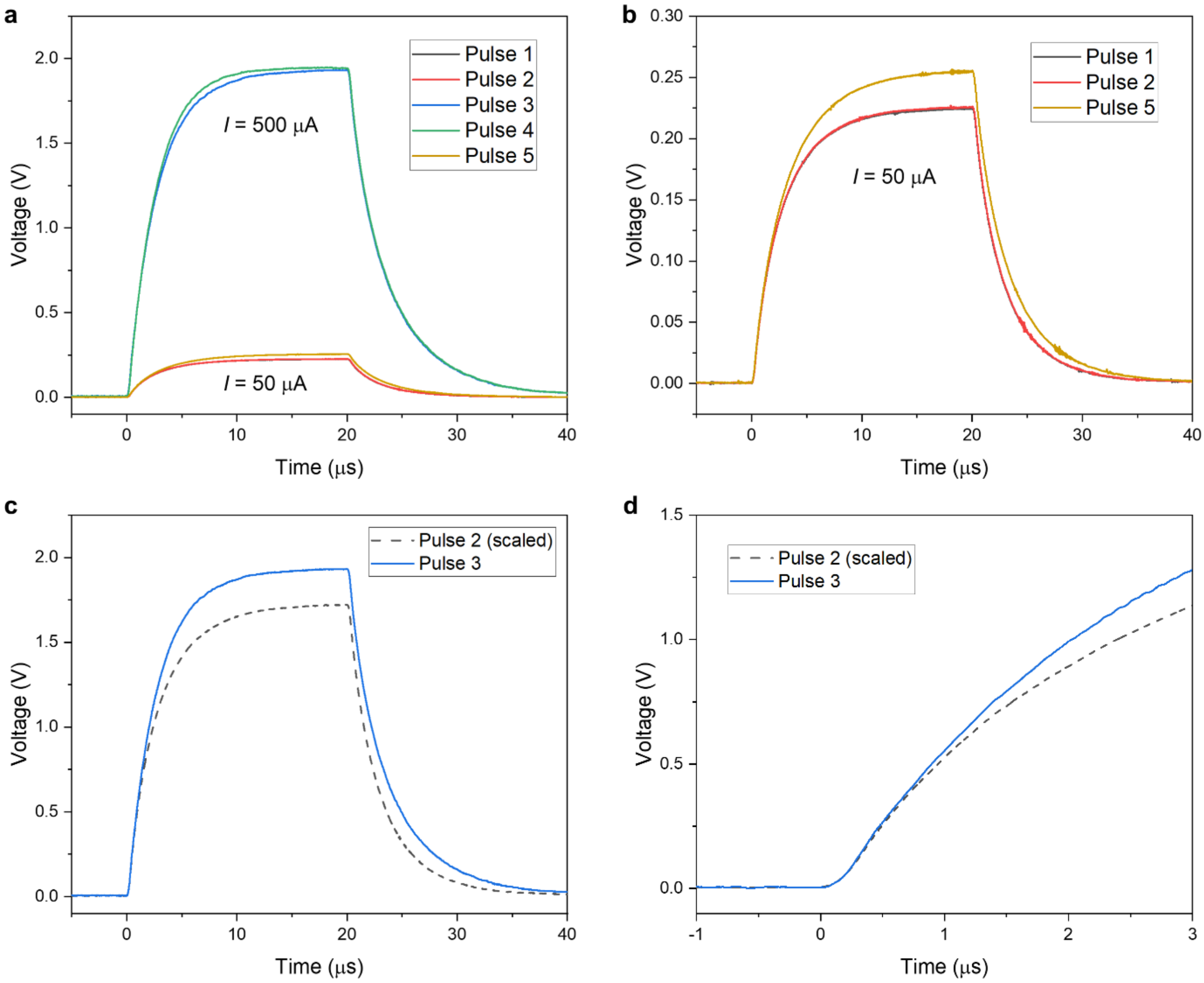

Supplementary Fig. 7. A sequence of current pulses (1-5) is applied while the voltage is monitored by an oscilloscope. **a**, Voltage traces of all pulses applied to the device. **b**, Close-up view of the pulses with 50 μA amplitude. **c**, Direct comparison of scaled pulse 2 (simulated response of no resistance change) to direct observation of pulse 3. The difference between the two is due to resistance switching during the pulse. **d**, Close-up view of the first few microseconds after the pulse onset shows that resistance increase happens on a sub-microsecond timescale – currently limited by the pulse rise time.

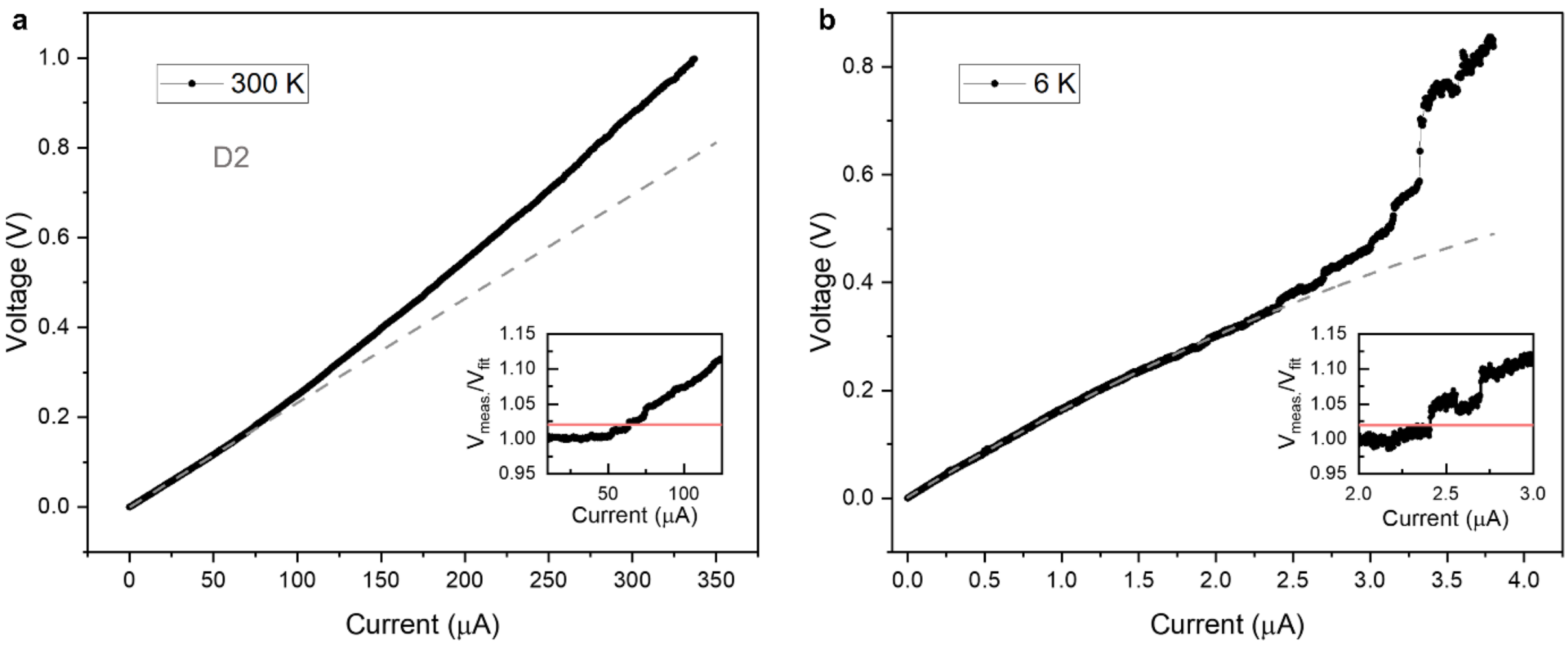

Supplementary Fig. 8. a,**b,** Pulsed *I*-*V* characteristic of device D2 at 300 K and 6 K, respectively. The dashed line is a fit to the low-voltage region. The inset shows the relative deviation from the fit, with the red line indicating the 2 % threshold.

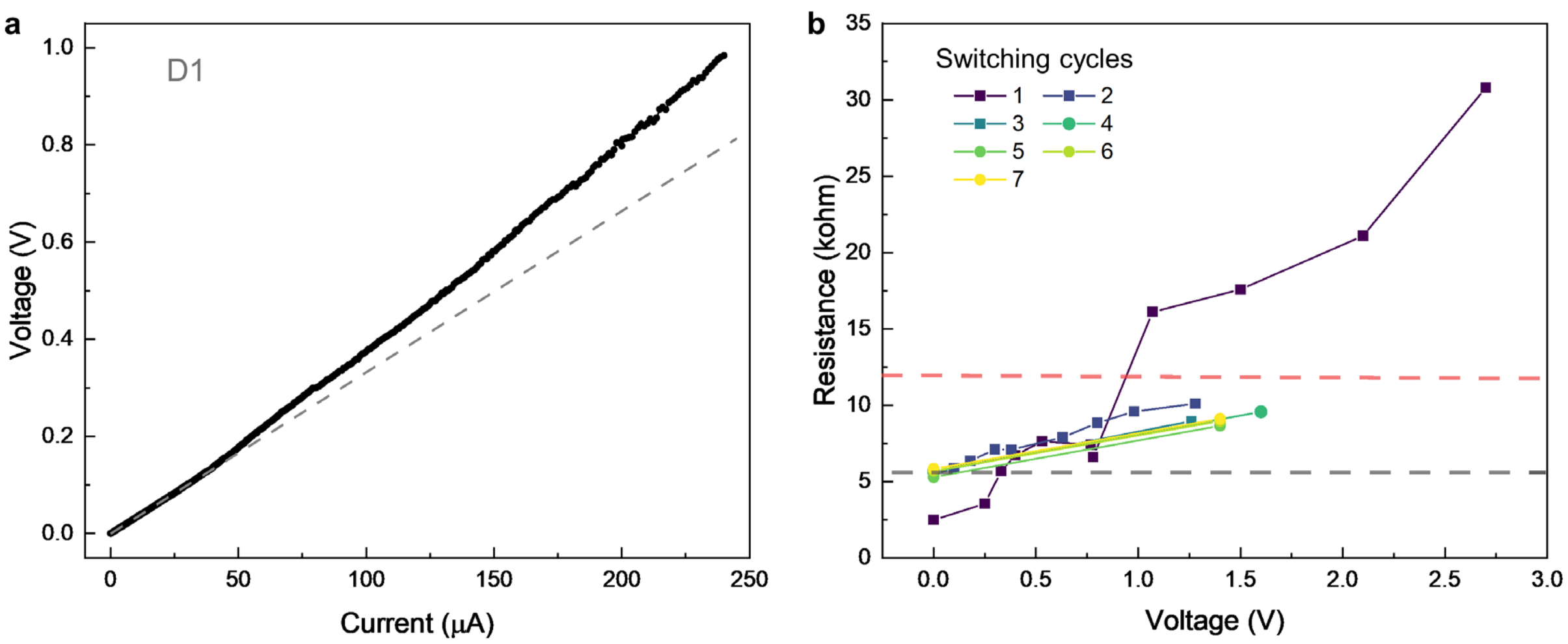

Supplementary Fig. 9. a Pulsed *I*-*V* characteristic. The dashed line is a linear fit to the low-voltage region. **b** Voltage dependence of resistance switching of all switching attempts on device D1 at 300 K. Switching attempts with a current source through outer electrodes (with voltage measured between inner electrodes) are marked with squares, while switching with a voltage source between inner electrodes is marked with circles.

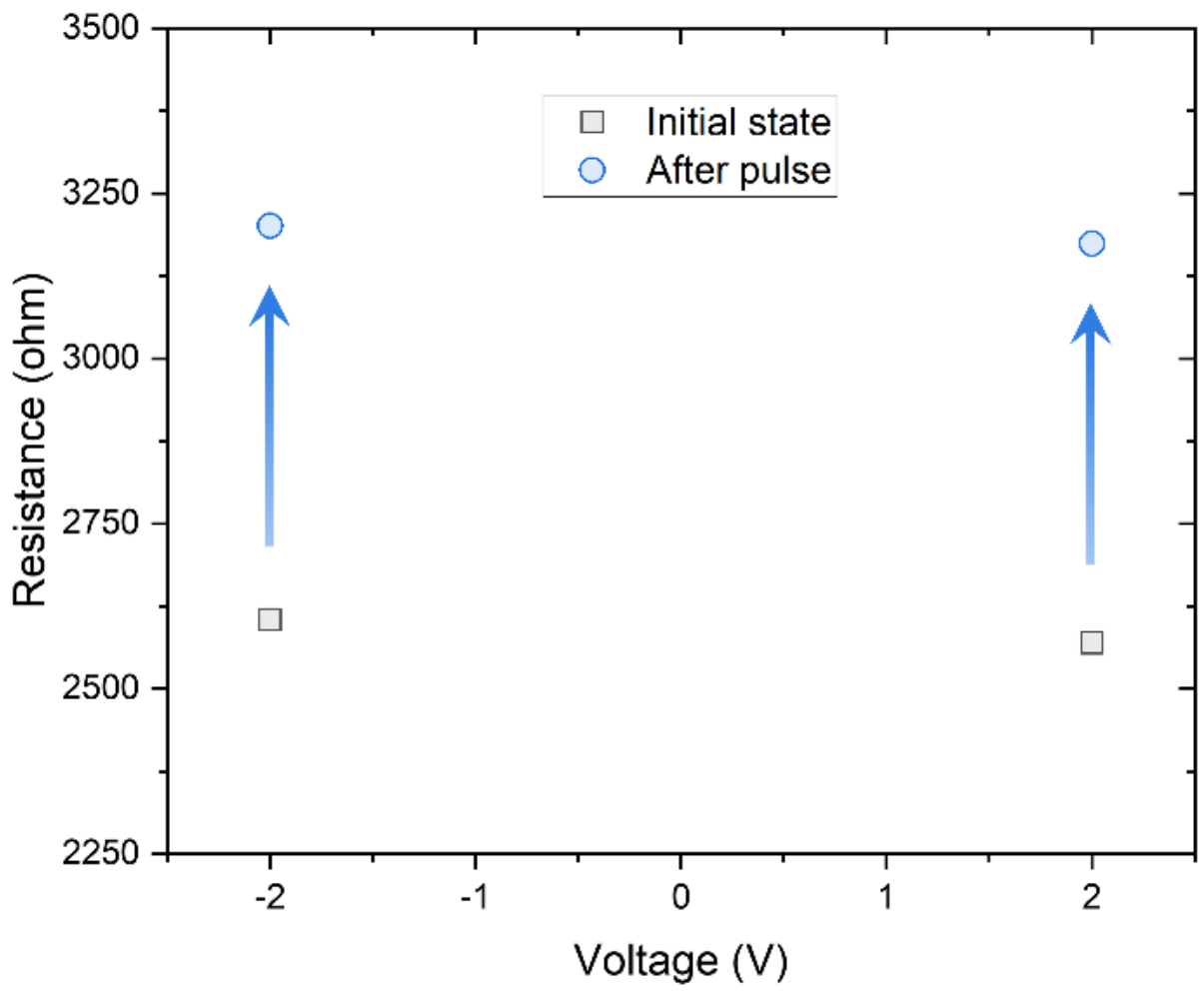

Supplementary Fig. 10. Resistance change of device D2 after 2.0 V electrical pulses of distinct polarities are applied to the cooling branch at 300 K.

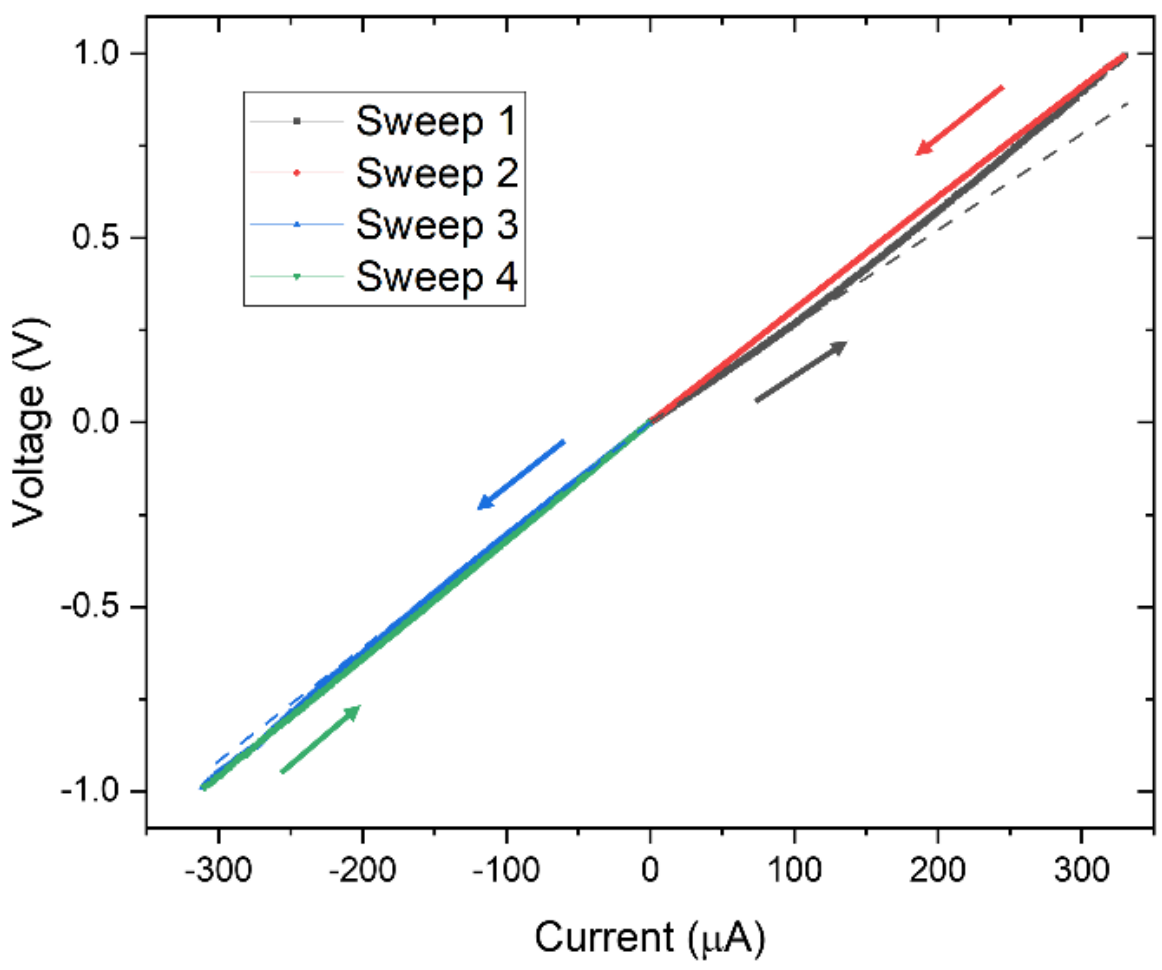

Supplementary Fig. 11. A sequence of pulsed *I*-*V* sweeps (from 1 to 4) applied to the cooling branch of the hysteresis at 300 K (device D2). Reversing the polarity of electrical pulses even slightly further increases the device resistance, instead of reversing the effect.

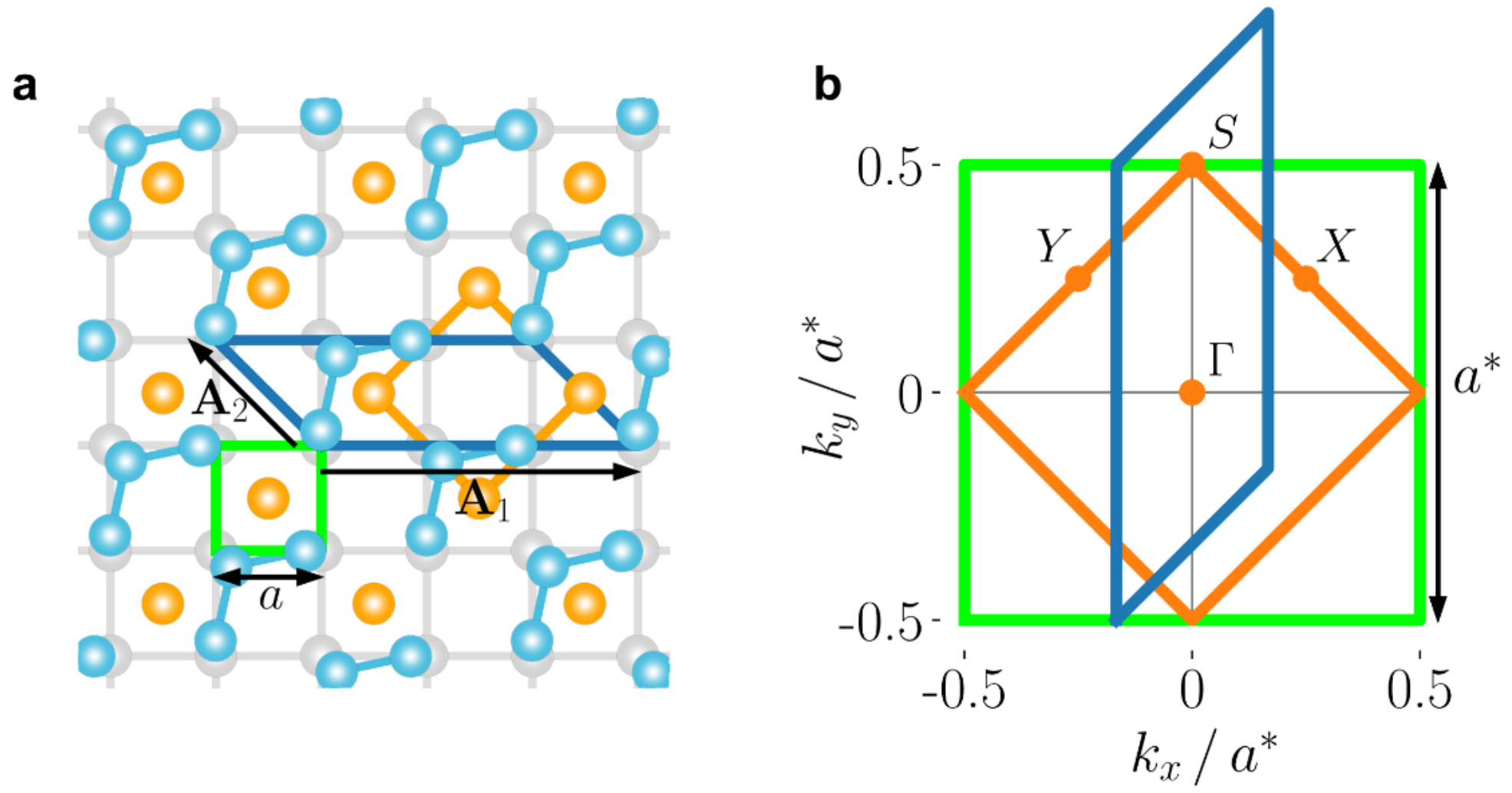

Supplementary Fig. 12. a) The real-space representation of a pure Te sheet (gray – undistorted, blue – distorted) with Eu atoms from the corrugated Eu-Te layer below shown in orange. The undistorted Te sheet unit cell is shown in green. The unit cell of the in-plane CDW phase for the Te sheet is shown in blue with primitive vectors marked as A_1 and A_2. The in-plane unit cell of undistorted $EuTe_4$ is shown in orange. b) The first Brillouin zones corresponding to the real-space unit cells from (a) are shown using the same colors as in (a). The high-symmetry points of the 3D Brillouin zone are labelled as well.

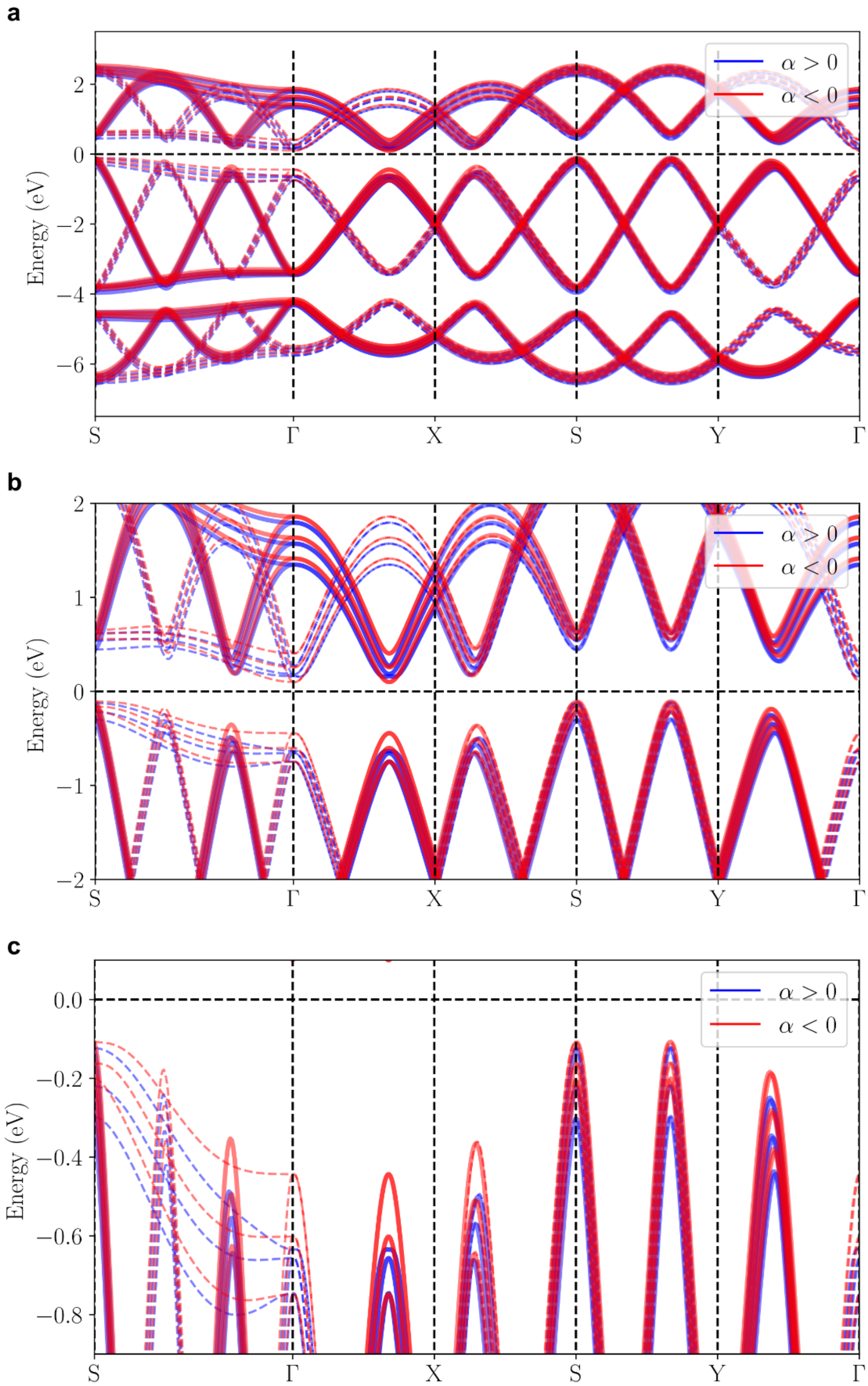

Supplementary Fig. 13 a-c. Electronic dispersions of the ground state (blue) and metastable state (red) along a high symmetry path in the 3D first Brillouin zone. For the ground state, the distortion of the third layer is $\alpha = +0.05$, while in the metastable state $\alpha = -0.05$, see text for details. From top to bottom, the same band structure is zoomed in. The thick lines correspond to bands inside the 3D FBZ, while the dashed bands are backfolded from the 2D to the 3D FBZ (see Supplementary Fig. 12b).

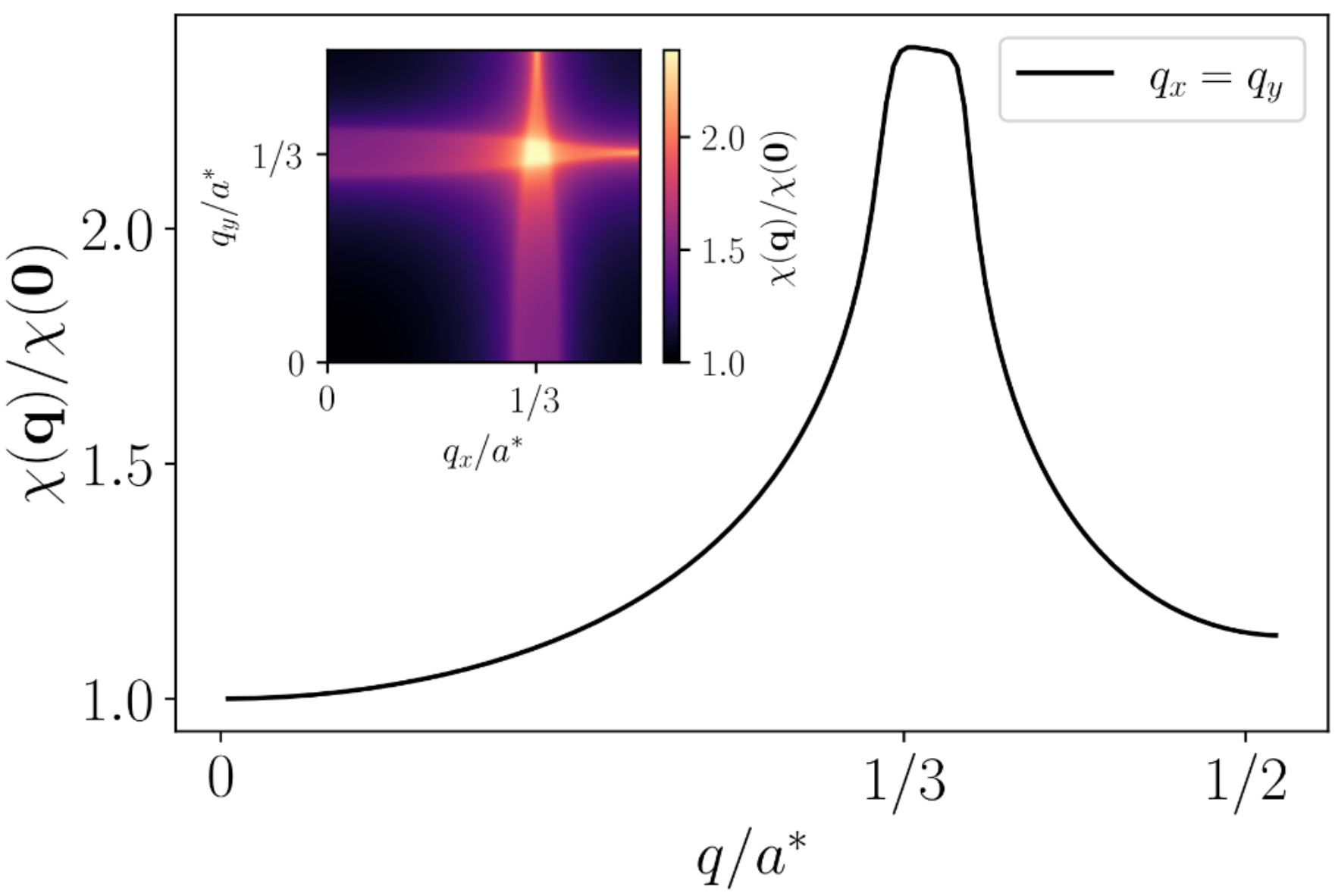

Supplementary Fig. 14. The bare susceptibility response of an undistorted square lattice monolayer with the electronic dispersion from the main text. The inset shows the response over a wider section of momentum space, while the main part of the figure shows the cut along the line $q_x = q_y$.

Supplementary Note 1: Shot-by-shot switching sequence (effect of pulse accumulation)

In Supplementary Fig. 1 we show the effect of applying a sequence of switching pulses of the same magnitude to the device D1 at 300 K. There is around 1 min between each pulse. We see that after initial switching with the first pulse, there is no accumulation effect of additional resistance switching due to adding more pulses to the switched state. This means that the state is settled after the first pulse is applied and is determined by the pulse magnitude.

Supplementary Note 2: Thermal cycle starting at 300 K from different initial states

In Supplementary Fig. 2 we show how an erase/heating thermal cycle influences the device for different initial states. The starting states are the cooling branch (Supplementary Fig. 2a), the heating branch (Supplementary Fig. 2b), or the electrical pulse switched state (Supplementary Fig. 2c). The experiments were done on the device D1.

By performing the thermal cycle from the cooling branch, we do not see a significant change in the resistance of the device, if anything, a minor lowering of the device's resistance. The thermal cycle from the heating branch reduces the device resistance, as expected from the hysteresis loop known for this compound. A similar trend is observed for the resistance of the device after switching.

We see that heating thermal cycles result in a lowering of the device resistance, irrespective of the starting state. Furthermore, the device resistance monotonously decreases with the increase in

temperature. This shows that the effect of the electrical pulse, which *increases* the resistance is different from any thermal effects.

Supplementary Note 3: Details of the pulsed *I*-*V* response at 300 K

Here we show the details of pulsed *I*-*V* measurements, which are done by incrementally increasing/decreasing current pulses while the voltage is measured during the pulse. Measurements are performed on the cooling branch at 300 K. The pulses are 250 μs long with a low duty cycle to reduce any thermal effects. As always, the measurements are performed in a 4-point configuration. The first sweep of increasing current pulses is the same measurement as shown in Fig. 2b.

In Supplementary Fig. 3a, we extend the measurement with the sweep of decreasing current. We perform a linear fit to the low-voltage section between 0 and 0.1 V (grey and blue dashed lines). While the black curve shows a significant deviation from the linear fit due to resistance switching, the linear fit describes the sweep-down curve well. In Supplementary Fig. 3b, we extend the measurement in a) by an additional sweep-up measurement. The pulsed *I*-*V* measurement closely follows the sweep-down measurement.

These measurements tell us that $EuTe_4$ has a linear *I*-*V* response (up to a moderate voltage of 1.0 V) in the absence of resistance switching. Furthermore, the state is determined by the highest voltage put on the device and resistance does not change further by applying lower voltage pulses (as demonstrated by the second sweep-up).

Supplementary Note 4: Pulsed *I*-*V* response by starting from either the cooling or heating branch

Next, we compare the pulsed *I*-*V* measurement by starting either from the cooling or heating branch at 300 K. Supplementary Fig. 4a is the same as Fig. 3b. Data shown in Supplementary Fig. 4b was recorded after the sample was cooled to 30 K and then heated up to 300 K. The pulsed *I*-*V* shows a sub-linear response, the opposite of the *I*-*V* curve measured on the cooling branch. The reverse sweep shows linear behavior, the same as on the cooling branch. This confirms that the sublinear behavior of the pulsed *I*-*V* is due to a resistance drop and not due to a device non-linear response or device heating. Furthermore, such a drop in resistance observed during the pulsed *I*-*V* measurement is consistent with the low-voltage regime in Fig. 2a. Again, as in Fig. 2a, the absolute value of deviation from linearity is larger for the cooling branch than for the heating branch.

Supplementary Note 5: Calculation of the upper boundary of energy for switching

Given that the switching behavior appears independent of the pulse length (See Fig. 2c), the leading edge of the pulse is likely responsible for the switching process. Therefore, by using short electrical pulses, the energy deposited in the device during the switching process can be exceptionally low. The shortest pulses that can be sourced to the sample without significant attenuation are 30 ns long. Supplementary Fig. 5a shows a 30 ns long pulse applied to the sample that is recorded by an oscilloscope. Supplementary Fig. 5b shows the change in resistance after the pulse is applied. The total pulse energy deposited to the device is calculated from the voltage transient $U(t)$ and resistance R of the sample with the equation $E = \int \frac{U^2(t)}{R} \mathrm{d}t$. The pulse is applied between the inner two electrodes, so the relevant resistance is 2-point resistance between the inner contacts, which we

measure before the pulse to be R = 4944 ohm (after the pulse: 5698 ohm). We calculate the total energy as

$$E = \int_{-20\ \mathrm{ns}}^{100\ \mathrm{ns}} \frac{U^2(t)}{R} \mathrm{d}t = 13\ \mathrm{pJ}$$

The value of 13 pJ is an upper boundary and overestimates the energy for switching for several reasons. Firstly, integration over the total pulse length overestimates the switching energy as only the leading edge of the pulse is likely relevant for the switching process. Additionally, an increase in the resistance during the switching process lowers the current through the device, which further lowers the energy dissipation. Finally, as the contact resistance is comparable to the $EuTe_4$ resistance, a drop in voltage on $EuTe_4$ itself is around two times lower compared to the value recorded on an oscilloscope.

Next, we can use the dissipated energy of the electrical pulse to estimate the change in temperature of the $EuTe_4$ device during the switching process.

$$\Delta T = \frac{E \times M}{C_p \times \rho \times V} = \frac{13\ \mathrm{pJ} \times 662.36\ \mathrm{g/mol}}{124 \frac{\mathrm{J}}{\mathrm{molK}} \times 6.711 \frac{\mathrm{g}}{\mathrm{cm}^3} \times 1.554\ \mu m^3} = 6.6\ \mathrm{K}$$

Where E is the energy of the pulse, M is the molar mass, C_p is the molar heat capacity, ρ is density and V is the the volume of the $EuTe_4$ flake between the electrodes (2 µm x 4.2 µm x 0.18 µm = 1.554 μm^3). The change in temperature of 6.6 K is an upper boundary as a more involved calculation would capture finite thermal coupling of the $EuTe_4$ flake to the substrate and electrodes, reducing the temperature reached.

In the calculation above, we take the relevant volume of the device during the switching process to be the whole $EuTe_4$ flake between the electrodes. While filamentary switching is common in some emerging memory devices (e.g., 1*T*-TaS_2 where a low-resistance channel forms), our observations do not demonstrate such a mechanism in $EuTe_4$.

For filamentary switching, a narrow, low-resistance channel appears in a device. In such cases, the appearance of a low resistance channel drives a large current through the filamentary path, causing localized heating. Crucially, in $EuTe_4$ devices, the resistance increases during the switching.

If the switching were confined to a narrow filament transitioning to a high-resistance state, its impact on the device's total conductance would be minimal, as the current would continue to flow through the surrounding low-resistance part. The observed large increase in total resistance is therefore inconsistent with a filamentary process and indicates that a majority of the device volume participates in the transition.

Our experiments indicate that the current flow is reasonably uniform across the device. A strongly non-uniform or filamentary current flow would lead to significant localized Joule heating. Given the semiconducting nature of $EuTe_4$, this would cause a substantial reduction in the device resistance during the pulse. This would be directly observable in our pulsed I-V measurements as a sub-linear I-V response, which is inconsistent with our measurements. The absence of this sub-linear response provides strong evidence that significant localized heating does not occur, which in turn suggests that the current flow and subsequent switching are reasonably uniform across the device.

Supplementary Note 6: Reversible switching with electrical pulses

In Supplementary Fig. 6 we show that it is possible to erase the switched state (and perform reversible switching) also by applying high-current electrical pulses. Unlike measurements shown in Fig. 2 of the manuscript, pulses here are applied at a high temperature of 400 K, where we already observed the effects of heating for high-current electrical pulses as shown in Fig. 3c. At higher temperatures, we expect that even a smaller temperature change would lead to relaxation of metastable states. The pulse length is 200 µs for both 300 µA (write) and 480 µA (erase) pulses. For the first 20 pulses, we alternate between low-current pulses that increase resistance and high-current pulses that lower the device resistance. After that, we apply a few pulses without alternation to show that resistance is stable if the pulse magnitude does not change.

These measurements show that at 400 K, only a modest amount of additional Joule heating from the electrical pulse (480 µA for 200 µs) is needed to observe relaxation of the state (reduction of resistance). This is consistent with pulsed *I-V* measurements performed at 400 K (Fig. 3c), where device resistance increases due to electric field switching up until around 400 µA. For larger current values, the device resistance starts to decrease due to the Joule heating and an increase in the temperature of the device.

Supplementary Note 7: Time-resolved measurement of switching

Here we present the details of the time-resolved measurement of switching, the goal of which is to establish the time the device needs to settle into a high-resistance state following the pulse excitation. Supplementary Fig. 7a shows all five current pulses applied to the device in the cooling branch in sequence from 1 to 5. Pulses 1, 2, and 5 have a magnitude of 50 µA, while pulses 3 and 4 have a magnitude of 500 µA. Voltage during the pulse is detected via an oscilloscope. As electrical switching increases electrical resistance, we expect to see a transition to the novel state as an increase in voltage during the current pulse. As presented in Fig. 2, at room temperature resistance only gradually increases with an increase in voltage across the device. This means that we cannot expect any discontinuous features on the voltage trace that would indicate switching.

The voltage trace of the first two pulses applied (1 and 2) is almost identical, due to the low voltage which did not change the resistance of the device. Next, we apply two high-current pulses (3 and 4), followed by another 50 µA pulse (pulse 5). As seen in Supplementary Fig. 7b, the voltage during pulse 5 is significantly higher compared to pulses 1 and 2, which shows that the resistance of the device is now higher, and pulses 3 and 4 switched the device.

First, we point out that at the end of both high-current pulses (3 and 4), the voltage reaches the same value, which means that the device resistance at that time is the same. This immediately tells us that the device has already settled to a high resistance value during the pulse 3, which limits the upper limit for settling time to ~ 20 µs. However, due to the slow rise time of the circuit voltage slowly increases throughout pulse 3, which means that the resistance would increase a bit throughout the pulse even if the settling time is ultrafast.

To get a better understanding of how resistance changes during the switching pulse 3, we compare the voltage trace of pulse 3 to the hypothetical case of applying the same current pulse to the device without any resistance switching. To do that, we look at pulses 4 and 5, which we know did not change the resistance of the sample. The ratio of voltage amplitudes (V_4/V_5) corresponds to the voltage increase of the 500 µA pulse compared to the 50 µA in the absence of resistance change. Note that this value is a bit different from a ratio of currents due to a non-linear *I-V* response in a two-point measurement done here. The simulated response of a 500 µA current pulse with no resistance switching is therefore obtained by multiplying the voltage trace of pulse 2 by the ratio V_4/V_5.

The comparison of the simulated response to the 500 μA pulse without resistance switching to the experimental observation is shown in Supplementary Fig. 7c. A close-up comparison is shown in Supplementary Fig. 7d. From the comparison, we see that the change in resistance occurs already during the first microsecond of the pulse start. This means that the device settles to a higher resistance on a sub-microsecond timescale. The limitation to observing the effect on even shorter timescales is due to the slow rise time of the circuit. Note that while the pulse starts around time zero, the switching only initiates when the pulse voltage goes above around 0.2 V which is at around 0.5 μs. Also, due to low voltage within the first few microseconds of the pulse, the change in resistance is still small.

Supplementary Note 8: Determination of the switching threshold

As shown in Fig. 3, we observe an upward deviation in the *I-V* characteristic that signifies resistance switching across the whole explored temperature range. We set the threshold as a 2% deviation from the fit, which is a sufficiently large number to be robust with respect to noise and is yet close to the onset of the resistance change. For temperatures ranging from 100 K to 400 K linear fit captures the low-voltage region sufficiently. For low-temperature *I-V* measurements of 6 K and 30 K, we observe a sub-linear response at low voltages. This is easily explained via the Joule heating effect. The change in resistance with temperature is considerably steeper at lower temperatures, which means that already a small increase in temperature causes a significant change in device resistance which manifests as a sub-linear response. To capture this effect, the low-voltage region of the 6 K and 30 K pulsed *I-V* measurement is fitted by a second-order polynomial.

Supplementary Fig. 8 shows how the threshold for switching has been determined for 300 K and 6 K pulsed *I-V* measurements as an example of both high- and low-temperature responses. The inset shows a detailed view around the switching threshold with the red line indicating the 2% threshold.

To determine the threshold electric field for switching at 300 K for device D2, we take the threshold voltage determined in Supplementary Fig. 8a of 0.15V and divide it by the channel length of 2.0 μm to get the value of E_{th} = 0.75 kV/cm.

Supplementary Note 9: Resistance switching of device D1 at 300 K

In Supplementary Fig. 9, we show details on the switching behavior of device D1 at 300 K. Supplementary Fig. 9a shows a pulsed *I-V* characteristic of the device, with the onset of switching (deviation from linearity, again using 2% threshold as for device as in Supplementary Note 8) at around 0.12 V. The response is very similar to the characteristics of device D2. Given the threshold voltage of 0.12 V and a 1.4 μm channel size, the threshold electric field for device D1 is E_{th} = 0.86 kV/cm. This is comparable in size to D2, demonstrating consistent behavior.

Supplementary Fig. 9b shows all resistance switching attempts on device D1 performed at 300 K. After each switching attempt, we perform a heating cycle up to either 425 or 430 K. We see that after the first switching, the resistance baseline on the cooling branch at 300 K has increased, but has then remained stable on all following cycles of switching and heating. We have observed that the value of resistance on the cooling branch at 300 K is dependent on the value of the highest temperature reached during the heating cycle (higher temperature gives a state with lower resistance). It is likely that the initial state of D1 was similar to the state reached by going to higher temperatures. The subsequent heating cycles went up to 425 K or 430 K, and the resistance of the device remained stable. For device D2, all heating cycles went only up to 425 K. While the resistance on cooling branch at 300 K remained very stable, we occasionally observe its change after a heating cycle. Overall, the resistance switching of device D1 is consistent with the behavior of device D2.

Supplementary Note 10: Polarity reversal of the electric field

Here, we present data on the polarity reversal of the electric field. As shown in Supplementary Fig. 10, we apply 2.0 V electrical pulse of both positive and negative polarity on the pristine state on the cooling branch. We do not observe a significant difference in switching.

Next, again on device D2, we perform a systemic investigation with pulsed *I-V* sweeps as shown in Supplementary Fig. 11. The sequence of sweep scans is performed in order from 1 to 4. For the first sweeps 1 and 2 we see a typical switching behavior of an increase in resistance. The resistance increases from the initial 2.61 kohm, to 3.06 kohm. Next, we perform a similar pulsed *I-V* sweep but in reverse direction, up to -1 V. After the sweep 4 is complete, we observe a moderate increase in resistance up to 3.21 kohm. Therefore, applying the pulse with negative polarity does not reverse the effect of switching with positive polarity. This shows that the effect of the electric field on the CDW is more complex and different from the behavior of ferroelectric materials.

Supplementary Note 11: Unit cells, Brillouin zones and electronic dispersions

The theoretical framework that we present involves only the nearly square sheets of pure Te in $EuTe_4$. For these, we opt for a more convenient description based on a 2D square unit cell (green in Supplementary Fig. 12) rotated by 45° and scaled down by a factor of $\sqrt{2}$ with regard to the actual in-plane unit cell of $EuTe_4$ (shown in orange in Supplementary Fig. 12). The 2D lattice constant a is thus related to the in-plane lattice constants $\tilde{a}, \tilde{b}$ via $\tilde{a}, \tilde{b} \approx \sqrt{2}a$. In Supplementary Fig. 12, we show the various relevant unit cells, both in real (Supplementary Fig. 12a) and momentum space (Supplementary Fig. 12b).

For completeness, we provide in Supplementary Fig. 13 the fully gapped electronic dispersions of both the ground and metastable minimum from the computations presented in Fig. 4a. These are shown along a high-symmetry path in the 3D first Brillouin zone (orange in Supplementary Fig. 12). The dashed bands are obtained by backfolding the "corners" of the 2D first Brillouin zone (FBZ), shown as green in Supplementary Fig. 12, back into the 3D FBZ via a translation by a wave vector equivalent to the side of the 3D FBZ. The gap of the ground state dispersion (blue) is 568 meV, while the gap of the excited state dispersion (red) is 543 meV. We emphasize that we employed rough estimates of the microscopic parameters of our model, so while a qualitatively significant difference in the gap size is observed, the exact magnitude of the computed gap is subject to future investigation.

In the minimization procedure, we observed that the values of distortions for each layer are $\alpha = (0.05, 0.05, 0.05)$ for the ground state and $\alpha = (0.05, 0.05, -0.05)$ for the metastable states, respectively. In the ground state, the phases of the order parameter in each layer are $\theta_x \approx (0, 2\pi/3, 0)$ and $\theta_y \approx (2\pi/3, 0, 2\pi/3)$. In the metastable state, the phases of the order parameter in each layer are $\theta_x \approx (0, 2\pi/3, 5\pi/3)$ and $\theta_y \approx (2\pi/3, 0, \pi)$.

Supplementary Note 12: Susceptibility response of the tight-binding model

We observe that the in-plane periodicity of the incommensurate CDW phase of $EuTe_4$ deviates little from a commensurate phase with a CDW wave vector $\mathbf{q} = \left(\frac{1}{3}a^*, \frac{1}{3}a^*\right)$. The susceptibility response (generalized Lindhard function) of the tight-binding model presented in the main text exhibits a pronounced peak at that point (Supplementary Fig. 14). In our theoretical framework, we therefore approximate the ground state with the commensurate situation.
