# Supplementary material for: Room-temperature memristive switching between charge density wave states
Source: Nat Commun. 2026 May 21;17:8360. doi: 10.1038/s41467-026-73267-x (PMC13473091; doi:10.1038/s41467-026-73267-x)
Supplement: Supplementary file 2 [file 41467_2026_73267_MOESM2_ESM.pdf]

nature portfolio

Peer Review File

Room-temperature memristive switching between charge density wave states

Corresponding Author: Dr Rok Venturini

This file contains all reviewer reports in order by version, followed by all author rebuttals in order by version.

Version 0:

Reviewer comments:

Reviewer #1

(Remarks to the Author)
In recent years there have been significant advancements in manipulating the physical properties of quantum materials using various stimuli in condensed matter and material sciences. While ultrashort laser pulses have been successfully applied to induce phase transitions via nonthermal pathways in different materials, the use of electrical pulses for persistent/nonvolatile switching of electronic states is rare and has been limited to cryogenic temperatures. In this study, the authors have successfully used electrical pulses to control the properties of the layered CDW compound $EuTe_4$, across a wide temperature range from 6 K to 400 K. They discovered that electric pulses can excite hidden electronic states nonthermally, leading to non-volatile resistance switching. This switching is reversible through a thermal erase procedure. This work is of significance for the field and is expected to attract broad interest. Therefore, we recommend publication of the manuscript in Nature Communications. Nevertheless, we advise the authors to take into account and respond to the following comments prior to its acceptance.

Major comments:
Issues of E-field-driven dynamics. The manuscript's final section develops a theoretical framework based on Peierls' theory incorporating both interlayer stacking and its coupling to in-plane electronic ordering. The modeling “reveals the importance of coupling between the in-plane and out-of-plane orders that gives rise to the complex free-energy landscape capable of hosting long-lived metastable states observed in switching experiments”. However, the authors do not delve into the analysis of potential electric-field-driven dynamics, particularly how applied fields could guide the system through various metastable manifolds. Despite the theoretical challenge posed by the complex interaction landscape and multidimensional free-energy surface, the absence of theory-experiment correlations is somewhat regrettable. Incorporating additional analysis of E-field-driven dynamics, even if only approximate, would strengthen the final discussion in the manuscript.

a/b-axis anisotropy. In $EuTe_4$, the CDW vector q aligns with the b-axis, while the lattice distortion occurs along the a-axis. So, during the experiments by the authors, did the system exhibit anisotropic responses when electric fields were applied along different crystallographic directions? Given the bistable nature of Te-layer distortions (Fig. 4b,c) in the staggered model, could the resistance switching demonstrate direction-dependence relative to the applied electric field? For these issues, more experimental results are valuable, such as (1) Conducting a comparison of switching thresholds for E along a-axis vs b-axis fields. (2) Probing possible nonvolatile state control via field-direction protocols, or (3) Mapping the orientation-dependent switching phase diagram.

Furthermore, regarding the reversible switching issue, how does the resistance evolve when positive and negative above-threshold electric pulses are applied sequentially? Can complete all-electric resistance switching between multiple states be achieved through such field-polarity alternation?

Polar & CDW interplay. A crucial feature of $EuTe_4$ is its spontaneous polarization associated with CDW formation (Ref. 39 in the manuscript). Due to the semiconductor nature of $EuTe_4$, this polarization should exhibit pronounced sensitivity to external E- fields. In addition, the direction-dependent CDW stacking configurations (Figs. 4b,c) generate various polarization states, suggesting a highly anisotropic and potentially strong asymmetric E-field response. Various experimental approaches could be employed to probe these direction-dependent responses. E.g., direction-dependent I-V curves - applying E // a and E // b to map out polarization switching thresholds; or performing positive-up-negative-down measurements to distinguish possible polar/coercive characteristics [APPLIED PHYSICS LETTERS 98, 192901 (2011)].

SHG measurements would also be valuable for distinguishing between various field-driven metastable states, as their symmetry-dependent sensitivity could resolve subtle structural differences.

The band structure. The theoretical section states that 'the band structure of the monolayer CDW state is now completely gapped.' Since a full gap facilitates electric-field control by reducing free-carrier screening, we suggest including the detailed band structure (at least in Supplementary Information) to substantiate the key field-control claim and enhance the paper's completeness.

Minor comments:
In the manuscript Fig.3.e-"Temperature dependence of the threshold switching voltage (purple) and current (green)", the voltage should be "green", while the current "purple".

The notation Fig.4.c,d, should be Fig.4.d,e.

Reviewer #3

(Remarks to the Author)
Room-temperature memristive switching between charge density wave states

There is a current strong interest in 2D CDW materials, such as 1T-TaS2, that have shown interesting physical properties. The high temperature of the CDW transitions creates potential for practical applications, including memory and switching. The authors extended this line of research to EuTe4, a recently discovered layered material with CDW phases. The results are interesting. The authors offer a rigorous analysis of their experimental findings. I can recommend the paper for acceptance after the authors address the issues below.
1) What are the dimensions of the devices (lateral and thickness)? I was unable to easily find this information.
2) The authors present modeling results and briefly describe the theory in the methods. How was the incommensurate phase (without translation symmetry) treated computationally? Some comments on this (perhaps with a relevant ref) would help in understanding.
3) Referencing prior work on a switching in a related material 1T-TaS2 with applications in memory would benefit the readers (see "Electrical gating of the charge-density-wave phases in two-dimensional h-BN/1T-TaS2 devices", ACS Nano, 16, 11, 18968 (2022); Appl. Phys. Rev., 10, 041401 (2023)).
4) The authors mention Joule heating in several places. Is it possible to provide estimates for the temperature rise due to Joule heating (self-heating) for their devices?

Reviewer #4

(Remarks to the Author)
The authors present a study of a layered CDW material, EuTe4, which exhibits several distinct CDW orders, and a hysteretic behavior in the resistivity as a function of temperature. The authors show that by applying voltage pulses they can drive the material into a different point on the resistivity curve, suggesting that this is a hidden state in the material of some sort. They go through some lengths to show that this is likely not a thermal effect, which seems quite reasonable based on their results. They also show that whatever they are seeing occurs rapidly, or at least shorter than they can resolve. The state the system is driven into appears to be stable for long times, and at non-cryogenic temperatures. They report that the system is likely modified by an electric field effect. Some theoretical analysis of the free energy in a restricted range is presented based on the Peierels/nesting model of CDW formation.

Overall, the experimental observations are worthwhile, and demonstrate interesting behavior of this material under new conditions. The theoretical analysis is fine, although I have some questions (below). Mostly, the manuscript does not make the case that this is some new state sufficiently clear. I agree with the authors that it is probably non-thermal, and the change in the resistivity is an indicator that something is different, but the conclusion that this is a non-thermal hidden state needs stronger backup than that. It could, for example, a more disordered version of the existing state, or some other disordered effect that has occurred due to the applied field.

On the theory side, the analysis seems oversimplified. There is nothing a priori wrong with it barring the question below, but it seems like this material is exhibiting something new. How is it then that it should be captured by a standard Peierls picture, which in any case should have picked up the thermal phases and others like it? Moreover, if the energy barrier is really just 15 meV, one would expect thermal fluctuations to appear quite readily, or at least to have some strong temperature dependence on that scale which seems to be absent in the data.

Thus, while I find this work interesting, I do not think it warrants publication in nature communications without a more thorough study of what the actual changes in the material are.

Minor points:

Do you have a sense as to how big the electric field is? Is it a sensible scale to cause the electrical switching in a CDW?

Fig 1 -- it is generally unclear which aspects of the figure are being referred to in the main text. For example, where is the jump in resistance due to the 2.7V pulse?

The discussion in comparison to the semiconductor is not very clear. I gather that the goal is to say that the switching is non-thermal because a semiconductor (which this ostensibly is based on the IV curve) shows sublinear behavior in the IV curve and decide D1 does not. Currently, it reads as if a comparison is made to say that D2 is not a semiconductor.

Finally, a figure of the Lindhard function at w=0 would be very helpful. How distinct is the peak? Rare-earth tritellurides typically do not show a sharp peak in the susceptibility (see e.g. Johannes and Marin, PRB2008). Fig 4 is not helpful because it has no proper scale.

The expression for the Lindhard response is odd. It appears that m,n index the bands and l,\alpha index the orbitals. Without the orbital sum, this is an orbitally resolved susceptibility. Why take the sum of all these elements? A measurable susceptibility is the trace. Please confirm that this is the appropriate susceptibility.

Reviewer #5

(Remarks to the Author)
In this work, Venturini and colleagues studied the electrical resistivity switching phenomena in a recently-discovered CDW material, EuTe4. The team found that the material can enter a metastable, higher-than-normal resistivity state following the application of electrical pulses, which is very stable if left unperturbed, but can return to the equilibrium state after thermal cycling. The authors presented convincing evidence that the electrical pulse-induced switching cannot be explained by a trivial joule heating scenario, and the switching occurs shorter than 30 ns. Quite remarkably, the switching is somewhat deterministic for both ways depending on the amplitude of the current pulse, as shown in Figure S6.

Overall, I find the results very interesting even though there has been an earlier demonstration of a similar phenomenon using ultrafast laser pulses in Ref. [39]. From an application standpoint, electrical switching at room temperature is much simpler and hence more appealing than the complex setup of an ultrafast laser. The author team also conducted a series of systematic experiments that aimed to constrain the possible mechanisms of this switching phenomenon, which, despite a lack of definite conclusions of the microscopic switching pathways, are still very valuable. However, I have a number of technical concerns, which may prevent readers from fully appreciating the significance of these results. I hence recommend a revised manuscript to be published in Nature Communications after the authors address the following points.

1. Although I am convinced about the non-thermal nature of the switching as explained in the first paragraph of the "Discussion" section on page 7, I have some reservation about the estimated 6.6 K (which is quoted as 7 K in the main text) temperature increase, which is calculated on page 5 of the Supplementary Information. In this 6.6 K estimate, the authors assume that the 13 pJ energy is uniformly distributed throughout the sample volume between the electrodes. However, for semiconductors, spatial distribution of current flow is rarely uniform, and in the extreme case, there can be a filamentary current flow. I would expect this non-uniformity to hold especially for EuTe4 if there are different metastable domains. Is there a way to show that the current flow is indeed uniform as the authors assumed? Or is there an alternative way to estimate an upper bound on the temperature increase of the region of the sample that switched, which is not necessarily the entire sample volume between the two electrodes?

2. Still on the point of joule heating, I find it surprising when comparing Figure 2c and Figure S6. In Figure 2c, the pulse length is varied by 8 orders of magnitude at a constant voltage pulse amplitude, meaning that the energy carried by the pulse also changed by 8 orders of magnitude. However, that has no effect on the final, electrically switched state. The authors interpreted this observation as evidence for a non-thermal switching behavior. In Figure S6, by changing the pulse amplitude from 300 μA to 480 μA while keeping the pulse duration the same at 200 μs, they can switch between a nonthermal "writing" pulse and a joule-heating "erase" pulse. This looks very strange: applying a 2.0 V pulse for as long as 1 second in Figure 2c still has no joule heating effect (estimated current is 2.0 V / 3200 Ω = 625 μA), but applying a 480 μA current for 200 μs in Figure S6 enters the joule heating regime.

3. The authors concluded from Figure 3e that "the electric field drives the switching to the novel states and not the current injection". I did not quite follow the physical reasoning here. Why does a constant voltage threshold in Figure 3e indicate that it is the electric field that drives the switching behavior? And can the author relate the voltage value to a threshold electric field, and discuss how the field strength relates to known microscopic parameters of EuTe4?

4. The authors mentioned the recent experiments in Ref. [39] about the laser-induced non-volatile switching in EuTe4. I think the readers will benefit from a more thorough discussion about the similarities and differences between the phenomenology of the present electrical-switching experiments and the previous laser-switching experiments.

5. Comparing the equilibrium R-T curves between Figure 3a and Figure 1d, besides the one-order-of-magnitude difference in the actual resistance (presumably due to sample size and contact resistance), the two hysteresis loops also look quite different. Can the authors comment on which phenomenology regarding the resistivity switching is sample dependent, and which is not? For instance, from Figure 3e, is the threshold electric field (need to convert voltage to field) at each temperature sample dependent? And is the percentage resistance increase under the same electric field the same across samples at a fixed temperature?

6. In the introduction paragraph on page 1, the authors mentioned “As [resistance switching in CDWs] is based on changes to the electronic instead of the structural order, such switching was shown to be ultrafast, ultraefficient, and has high endurance.” I am confused by this comment because doesn’t the periodic lattice distortion of the CDW (for both 1T-TaS2 and EuTe4) change when it enters a metastable state? For 1T-TaS2, the authors mentioned that there is a “network of CDW domains and domain walls”, and the atomic positions at those domains/domain walls are presumably modified in addition to electron density modification. Similarly, for EuTe4, the authors mentioned the out-of-plane arrangement of bilayers and monolayers of Te, and I would imagine that the Te ions have to move when going from the equilibrium state to a metastable state. In that case, the structural order is also involved.

7. The two-way resistance switch in Figure S6 is quite subtle, where the change in resistivity is very small for a semiconductor. Have the authors found a similar two-way resistance switching at cryogenic temperatures? Or is there a reason that the low-temperature switching is not two-way?

8. I find the theory discussion on page 7 to page 9 can benefit from a more rigorous exposition. I have a few comments:

(i) The adoption of Peierls’ description of the CDW in EuTe4 is rather simplistic and possibly wrong. The authors assumed a momentum-independent electron-phonon coupling g on page 12. For a closely related CDW material, RTe3, it has in fact been shown that anisotropic electron-phonon coupling, where g is both momentum and phonon-branch dependent, is the dominant factor; see Frank Weber’s inelastic X-ray scattering results in Phys. Rev. B 91, 235146 (2015).

(ii) The bigger question about the theory discussion is that it does not seem to show how and why the application of electrical pulses can switch the systems between different metastable states. The presented theory only shows the possibility for metastable states to exist, which is already known from previous experimental studies cited by the authors. I think what is missing here is

(a) the connection between the theoretically proposed metastable states to the different resistivities associated with these states,

(b) what happens microscopically when an electrical pulse passes through the sample, and

(c) how to reconcile different features of the switching phenomenology in Figures 1 to 3, such as it is electric field driven, it increases instead of decreasing the resistivity, and why the electrically-induced metastable states are different from the metastable states in the hysteresis loop.

(iii) As a minor note, it would be beneficial to draw the coordinate system when the authors switched to the q = (1/3a, 1/3a) notation on page 7 even though the in-plane CDW is along the b axis, as mentioned on page 2. I am afraid not all readers may catch the 45° rotation between the square net of the Te monolayer and the actual unit cell.

Version 1:

Reviewer comments:

Reviewer #1

(Remarks to the Author)
For our previous queries, the authors have provided detailed clarifications and arguments supported by additional experimental data and theoretical analysis.

The revised version appears more in-depth and rigorous. In general, again, this work demonstrates the non-thermal and nonvolatile memristive switching at room temperature in CDW EuTe4 induced by pulsed electric fields, which exhibits unique kinetic characteristics distinct from those of previously reported systems. Given the complex nature of the intrinsic interactions in this material, we agree that a complete understanding of the underlying dynamics will require further experimental investigation beyond the scope of a single article.

We have no further concerns regarding the revised manuscript and recommend its acceptance for publication.

Reviewer #2

(Remarks to the Author)
I co-reviewed this manuscript with one of the reviewers who provided the listed reports. This is part of the Nature Communications initiative to facilitate training in peer review and to provide appropriate recognition for Early Career Researchers who co-review manuscripts.

Reviewer #3

(Remarks to the Author)
The authors responded properly to reviewers' questions and requests. I recommend the acceptance of the revised version.

Reviewer #4

(Remarks to the Author)
I will defer to the other reviewers on the experimental aspects. I do agree with Referee #1 that an absence of theory-experiment correlations is regrettable, and that such an analysis would much improve the manuscript. In my estimation, this remains necessary before publication in Nature Communications, although the other reviewers disagree with me on this.

The authors have improved the description of the theoretical work performed, but it is still unclear. The issues below should be addressed and corrected before publication is warranted.

* There could be more clarity on the distinction between orbital and band indices in the manuscript. I gather that the two orbitals do not interact at all, but this is not clear.

* If the former is true, then why is there a matrix nature to the Hamiltonian? Is this just interlayer coupling, and the authors are considering two independent sets of bands?

* Reference is made to the Hamiltonian matrix h(k) on line 353, which is not defined, and its connection to the eigenvalues \varepsilon_m(k) -- a variable that also appears on line 330 -- is not clear. If h(k) is intended to mean the one that appears on line 375, then the ordering is incorrect.

* Line 346 appears like it is missing text.

* Please clarify what the "canonical displacement variables" (line 379) mean.

* The band structure in Fig S13 is quite strange. There are discontinuities just to the side of the high symmetry points of the BZ (not right at them); see e.g. the topmost red line crossing the X point or S point as an example. Moreover, if the reduced zone picture is correct, then the bands should approach the Brillouin zone edge with 0 slope. They clearly do not, which suggests they should be plotted in the larger zone.

* The Lindhard susceptibility (S14) does have a peak, but it is only marginally larger than the background. A momentum-dependent electron-phonon coupling does help to form a clear CDW with a well-defined peak, but I cannot tell from the formalism as outlined where that goes into the calculation. The vertex appears to have an orbital (band?) dependence, but the susceptibility expression does not. If the momentum (orbital/band) dependent vertex simply goes into the mean field CDW procedure that this is fine if clearly stated; it would then also be helpful have a Lindhart susceptibility that incorporates it to be consistent in what is being used for the condensation.

Reviewer #5

(Remarks to the Author)
I thank the authors for the revised manuscript and the point-by-point response to my and other referees' comments. While I stand by my previous assessment that the work would be valuable for publication in Nature Communications, there are still outstanding technical points that require additional attention. I list them below.

Concerning my Comment 3 in the previous round of review, I asked, "can the author relate the voltage value to a threshold electric field, and discuss how the field strength relates to known microscopic parameters of EuTe4?" The authors did not address this question in their response to Comment 3, but I found in their response to referee #4 that the threshold fields were approximately 0.75 and 0.86 kV/cm, and the value is comparable to the depinning field in 2D CDW materials such as 1T-TaS2. However, it is unclear to me what is the relationship between depinning — if it occurs in EuTe4 at all — and the resistive switching observed in their experiments. I think it will benefit readers if the authors can provide some intuition about the magnitude of the threshold field even if the authors cannot nail down an exact microscopic mechanism.

Concerning my Comment 8(ii)(b), which asks, "what happens microscopically when an electrical pulse passes through the sample?", the authors did not address the question. The same question is also raised by referee #1. While I understand the complexity of the exact physical scenarios involved, I think it would still be beneficial to provide some intuition about the possible scenarios or provide some constraints to exclude certain scenarios. For example, the fact that electric field polarity does not matter despite the CDW phase being polar can rule out certain scenarios where there is a direct dipolar coupling between the electric field and the polar lattice. A more thorough discussion can benefit interested readers in joining force to pin down the exact mechanism through future works.

Concerning Fig. S14 in the response letter (which is labeled as Fig. S12 in the Supplementary Information), the caption says "the unit cell of the in-plane CDW phase is shown in blue." This schematic is contradictory to the fact that the CDW is approximately tripling the unit cell only in the crystallographic b direction, so the in-plane CDW unit cell should be a rectangle that is 45-degree rotated.

Concerning Fig. S13, how were the electronic dispersions calculated? The authors should specify the value of alpha's used in the plot. It should be noted that the schematic electronic dispersions in Fig. S13 qualitatively contradicts to the measured electronic dispersion in angle-resolved photoemission spectroscopy, see for example, Zhang et al., Phys. Rev. B 106, L201108 (2022); Lv et al., Phys. Rev. Lett. 128, 036401 (2022); Zhang et al., Phys. Rev. B 107, 115141 (2023); Elius et al.

arXiv:2508.06464 (2025). The dispersions between Gamma-X and Gamma-Y are not symmetrical (see Fig. 2 in Zhang et al. 2023), and the authors should comment on how this major discrepancy impacts the tight-binding results on the electronic properties of the two CDW states.

Minor comment: On page 8 of the revised manuscript, the authors wrote "i.e., rotated by 45º w.r.t. the unit cell of $EuTe_4$ and scaled accordingly50". The reference [50], which concerns $BiFeO_3$ thin films, looks puzzling in this context.

Version 2:

Reviewer comments:

Reviewer #5

(Remarks to the Author)
The authors have addressed several technical points raised by me and Reviewer #4 in the previous round of review. In my opinion, the physical intuition of what causes the proposed out-of-plane stacking rearrangement is still insufficiently developed. Nonetheless, at this stage, the paper would better serve the community if published in Nature Communications. I hence recommend the acceptance of this revised version.

(Remarks on code availability)

Open Access This Peer Review File is licensed under a Creative Commons Attribution 4.0 International License, which permits use, sharing, adaptation, distribution and reproduction in any medium or format, as long as you give appropriate credit to the original author(s) and the source, provide a link to the Creative Commons license, and indicate if changes were made.
In cases where reviewers are anonymous, credit should be given to 'Anonymous Referee' and the source.
The images or other third party material in this Peer Review File are included in the article's Creative Commons license, unless indicated otherwise in a credit line to the material. If material is not included in the article's Creative Commons license and your intended use is not permitted by statutory regulation or exceeds the permitted use, you will need to obtain permission directly from the copyright holder.
To view a copy of this license, visit https://creativecommons.org/licenses/by/4.0/

Reviewer #1 (Remarks to the Author):

In recent years there have been significant advancements in manipulating the physical properties of quantum materials using various stimuli in condensed matter and material sciences. While ultrashort laser pulses have been successfully applied to induce phase transitions via nonthermal pathways in different materials, the use of electrical pulses for persistent/nonvolatile switching of electronic states is rare and has been limited to cryogenic temperatures. In this study, the authors have successfully used electrical pulses to control the properties of the layered CDW compound $EuTe_4$, across a wide temperature range from 6 K to 400 K. They discovered that electric pulses can excite hidden electronic states nonthermally, leading to non-volatile resistance switching. This switching is reversible through a thermal erase procedure. This work is of significance for the field and is expected to attract broad interest. Therefore, we recommend publication of the manuscript in Nature Communications. Nevertheless, we advise the authors to take into account and respond to the following comments prior to its acceptance.

Reply: We thank the reviewer for recommending our work for publication. We appreciate their view that the work is significant to the field and is expected to attract broad interest. We tried our best to follow the reviewer's suggestions and expanded our work with additional measurements.

Major comments:

Issues of E-field-driven dynamics. The manuscript's final section develops a theoretical framework based on Peierls' theory incorporating both interlayer stacking and its coupling to in-plane electronic ordering. The modeling "reveals the importance of coupling between the in-plane and out-of-plane orders that gives rise to the complex free-energy landscape capable of hosting long-lived metastable states observed in switching experiments". However, the authors do not delve into the analysis of potential electric-field-driven dynamics, particularly how applied fields could guide the system through various metastable manifolds. Despite the theoretical challenge posed by the complex interaction landscape and multidimensional free-energy surface, the absence of theory-experiment correlations is somewhat regrettable. Incorporating additional analysis of E-field-driven dynamics, even if only approximate, would strengthen the final discussion in the manuscript.

Reply: The nonequilibrium dynamics in the model proposed within this work is indeed an important next step and we have already taken steps along these lines. It turns out that the E-field response is rather complex and needs further work. Therefore, we would prefer to provide a detailed analysis as a follow-up to avoid misunderstandings due to a "quick-and-dirty" simplistic presentation.

a/b-axis anisotropy. In $EuTe_4$, the CDW vector q aligns with the b-axis, while the lattice distortion occurs along the a-axis. So, during the experiments by the authors, did the system exhibit anisotropic responses when electric fields were applied along different crystallographic directions? Given the bistable nature of Te-layer distortions (Fig. 4b,c) in the staggered model, could the resistance switching demonstrate direction-dependence relative to the applied electric field? For these issues, more experimental results are valuable, such as (1) Conducting a comparison of switching thresholds for E along a-axis vs b-axis fields. (2) Probing possible nonvolatile state control via field-direction protocols, or (3) Mapping the orientation-dependent switching phase diagram.

Reply: We agree that resolving the details of electric field interaction with the CDW order would be very valuable in improving our understanding of the switching behavior.

Probing the switching behavior along different crystal orientations and mapping the phase diagram [points (1) and (3)] presents significant experimental challenges. As the experiments are done on thin and microscopically small flakes, a different electrode structure and significant further efforts with photolithography would be needed to allow control of the direction of the electric field in a device. Furthermore, determining the crystal and CDW orientation for such small flakes requires advanced structural characterization, such as micro-focused X-ray diffraction at a synchrotron facility, which is currently beyond the capabilities of our laboratory. Given these substantial requirements, we believe a comprehensive study of anisotropy represents an important follow-up investigation that extends beyond the scope of this initial discovery.

While we cannot vary the in-plane field direction, we have addressed the question of electric field polarity dependence. To address this question, we present additional measurements, now added to the manuscript as Supplementary Note 10. Interestingly, our new data reveal that the observed switching appears to be independent of the electric field's polarity.

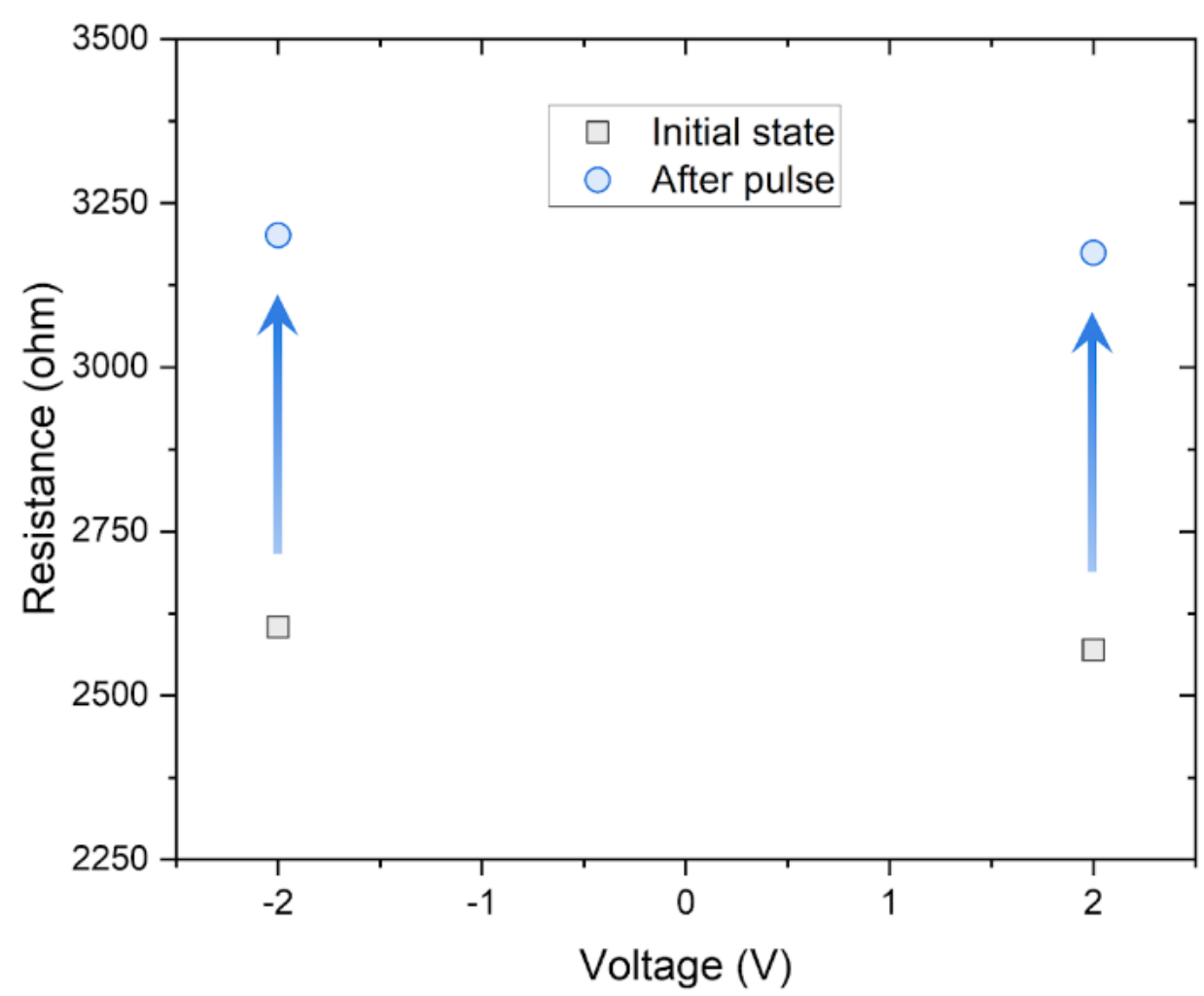

Fig. S10. Resistance change of device D2 after 2.0 V electrical pulses of distinct polarities are applied to the cooling branch at 300 K.

Furthermore, regarding the reversible switching issue, how does the resistance evolve when positive and negative above-threshold electric pulses are applied sequentially? Can complete all-electric resistance switching between multiple states be achieved through such field-polarity alternation?

Reply: As the reviewer suggests, we performed additional measurements on the device by applying electrical pulses of the opposite polarity on an already switched state (see Fig. S10, now shown in Supplementary Note 10). We observe that it is not possible to perform reversible switching by simply reversing the electric field. In fact, there is even a slight further increase in resistance upon applying pulses with reversed polarity. This shows that the observed electrical switching between CDW states in $EuTe_4$ is unique.

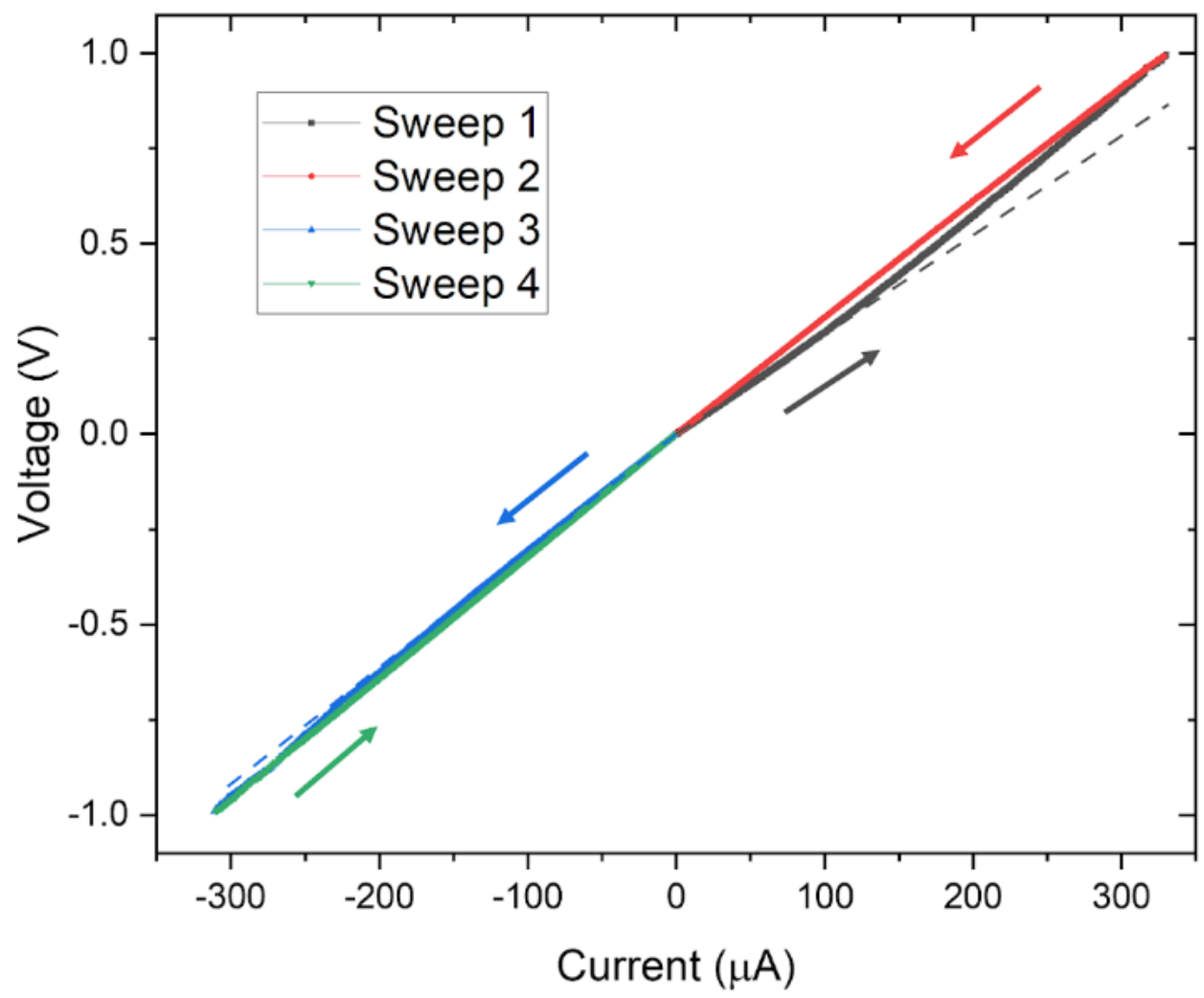

Fig. S11. A sequence of pulsed IV sweeps applied to the cooling branch of the hysteresis at 300 K.A positive pulse switches the device from a low to a high-resistance state (Sweeps 1 and 2). Subsequent negative pulses (Sweeps 3 and 4) do not reverse the switching but instead cause even a small further increase in resistance.

Polar & CDW interplay. A crucial feature of $EuTe_4$ is its spontaneous polarization associated with CDW formation (Ref. 39 in the manuscript). Due to the semiconductor nature of $EuTe_4$, this polarization should exhibit pronounced sensitivity to external E- fields. In addition, the direction-dependent CDW stacking configurations (Figs. 4b,c) generate various polarization states, suggesting a highly anisotropic and potentially strong asymmetric E-field response. Various experimental approaches could be employed to probe these direction-dependent responses. E.g., direction-dependent I-V curves - applying E // a and E // b to map out polarization switching thresholds; or performing positive-up-negative-down measurements to distinguish possible polar/coercive characteristics [APPLIED PHYSICS LETTERS 98, 192901 (2011)]. SHG measurements would also be valuable for distinguishing between various field-driven metastable states, as their symmetry-dependent sensitivity could resolve subtle structural differences.

Reply: Following the suggestion of the reviewer, we have performed additional IV measurements that exhibit direction dependence by inverting the polarity (see Fig. S11, now shown in Supplementary Note 10). We do not observe a substantial difference in the device's response to polarity reversal and it seems that along a certain atomic direction it is the magnitude of the electrical field that determines the state. We wish to emphasize here that the interplay of distinct CDW order in $EuTe_4$ is very complex and the effect of out-of-plane stacking is likely important, as has been pointed out in the recent work (Lv et al., arXiv:2501.09715). We agree that SHG measurements could be interesting, but due to the complexity of CDWs in $EuTe_4$, what we think is needed is a diffraction experiment that probes amplitudes of different CDW orders, including their out-of-plane component. We consider such experiments a promising route for future work.

We have added a discussion of these new results and their implications to the manuscript. The start of the 4th paragraph in the Discussion section, which now reads:

“Interestingly, resistance switching is independent of the pulse polarity, and we could not perform reversible switching simply by reversing the electric field (see Supplementary Note 10). This shows that the observed electrical CDW switching in $EuTe_4$ is very different from the reversal of polarization in ferroelectric materials [Wang et al., APL 2021, Chanthbouala et al., Nat. Nano. 2012]”

We also added a new Supplemental Note 10, discussing the effect of polarity reversal.

The band structure. The theoretical section states that ‘the band structure of the monolayer CDW state is now completely gapped.’ Since a full gap facilitates electric-field control by reducing free-carrier screening, we suggest including the detailed band structure (at least in Supplementary Information) to substantiate the key field-control claim and enhance the paper's completeness.

Reply: In agreement with reviewer comments, we add a figure of the electronic dispersion for both states corresponding to free-energy minima in Fig. 4a, along a high-symmetry path in the reduced Brillouin zone to the supplementary material and comment on it in the Discussion part of the main text.

“.. A comparison of the band structures for the two states shows that the state with $\alpha > 0$ always exhibits a larger band gap than the state with $\alpha < 0$ (see Fig. S13, now shown in Supplementary Note 11). Consequently, the resistivity of the former is also expected to be higher, which would be consistent with experimental observations. ..”

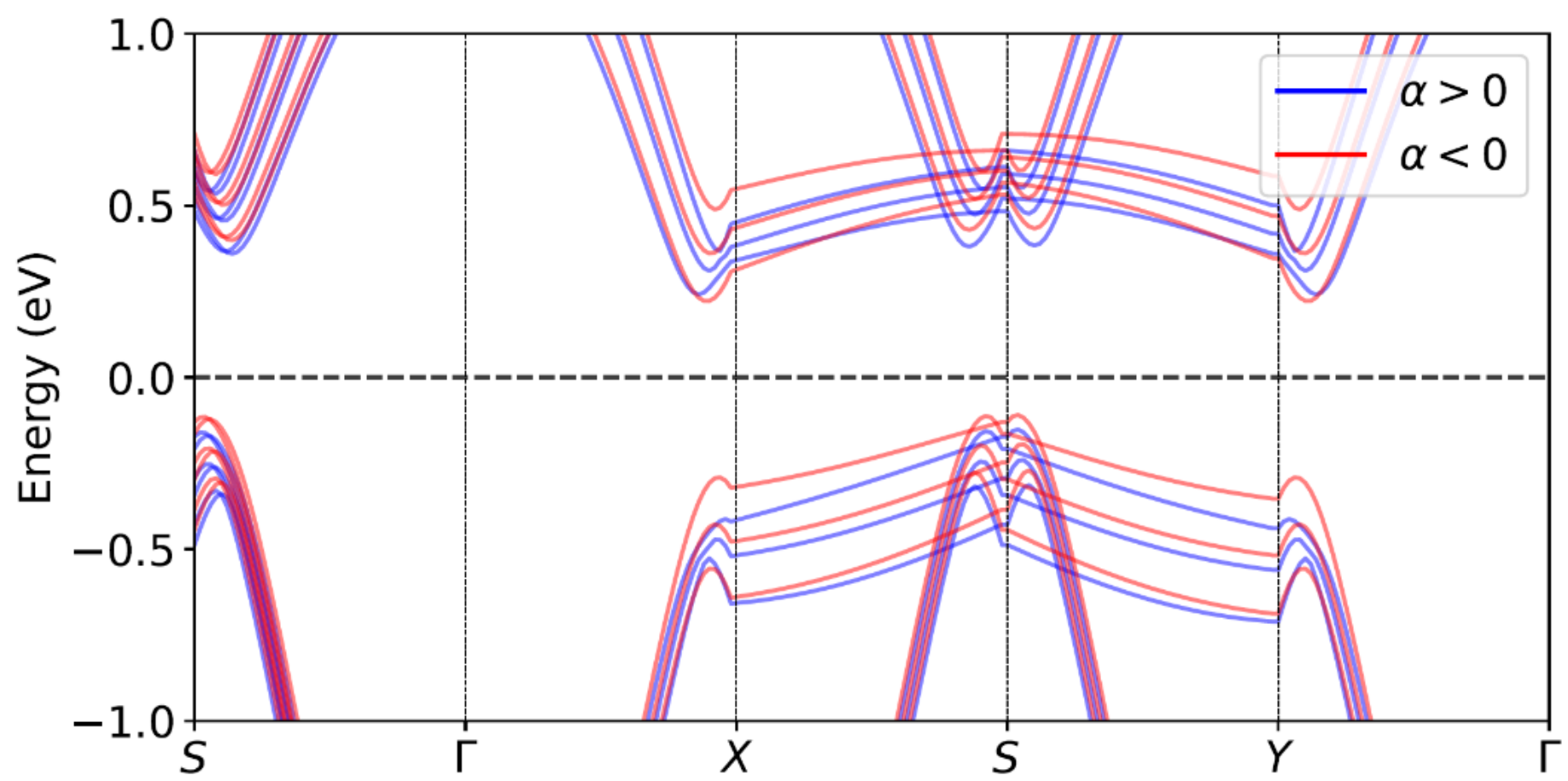

Fig. S13. Electronic dispersions of the ground state (blue) and metastable state (red) along a high symmetry path in the 2D reduced BZ.

Minor comments:

In the manuscript Fig.3.e-“Temperature dependence of the threshold switching voltage (purple) and current (green)”, the voltage should be “green”, while the current “purple”.

Reply: Thank you, fixed.

The notation Fig.4.c,d, should be Fig.4.d,e.

Reply: Fixed.

Reviewer #3 (Remarks to the Author):

Room-temperature memristive switching between charge density wave states

There is a current strong interest in 2D CDW materials, such as 1T-TaS2, that have shown interesting physical properties. The high temperature of the CDW transitions creates potential for practical applications, including memory and switching. The authors extended this line of research to $EuTe_4$, a recently discovered layered material with CDW phases. The results are interesting. The authors offer a rigorous analysis of their experimental findings. I can recommend the paper for acceptance after the authors address the issues below.

Reply: We thank the reviewer for describing our work as interesting and recommending its publication.

1) What are the dimensions of the devices (lateral and thickness)? I was unable to easily find this information.

Reply: Lateral size of device D1: flake width is 4.4 µm and gap between electrodes is 1.4 µm. Size of device D2: flake width is 4.2 µm and gap between electrodes is 2.0 µm. Device dimensions were determined via the AFM measurements. We have extended the Methods section with this information.

2) The authors present modeling results and briefly describe the theory in the methods. How was the incommensurate phase (without translation symmetry) treated computationally? Some comments on this (perhaps with a relevant ref) would help in understanding.

Reply: For the incommensurate phase, we evaluate the susceptibility response (generalized Lindhard function), which allows us to address the question of the CDW wavevector, see attached Figure S14.

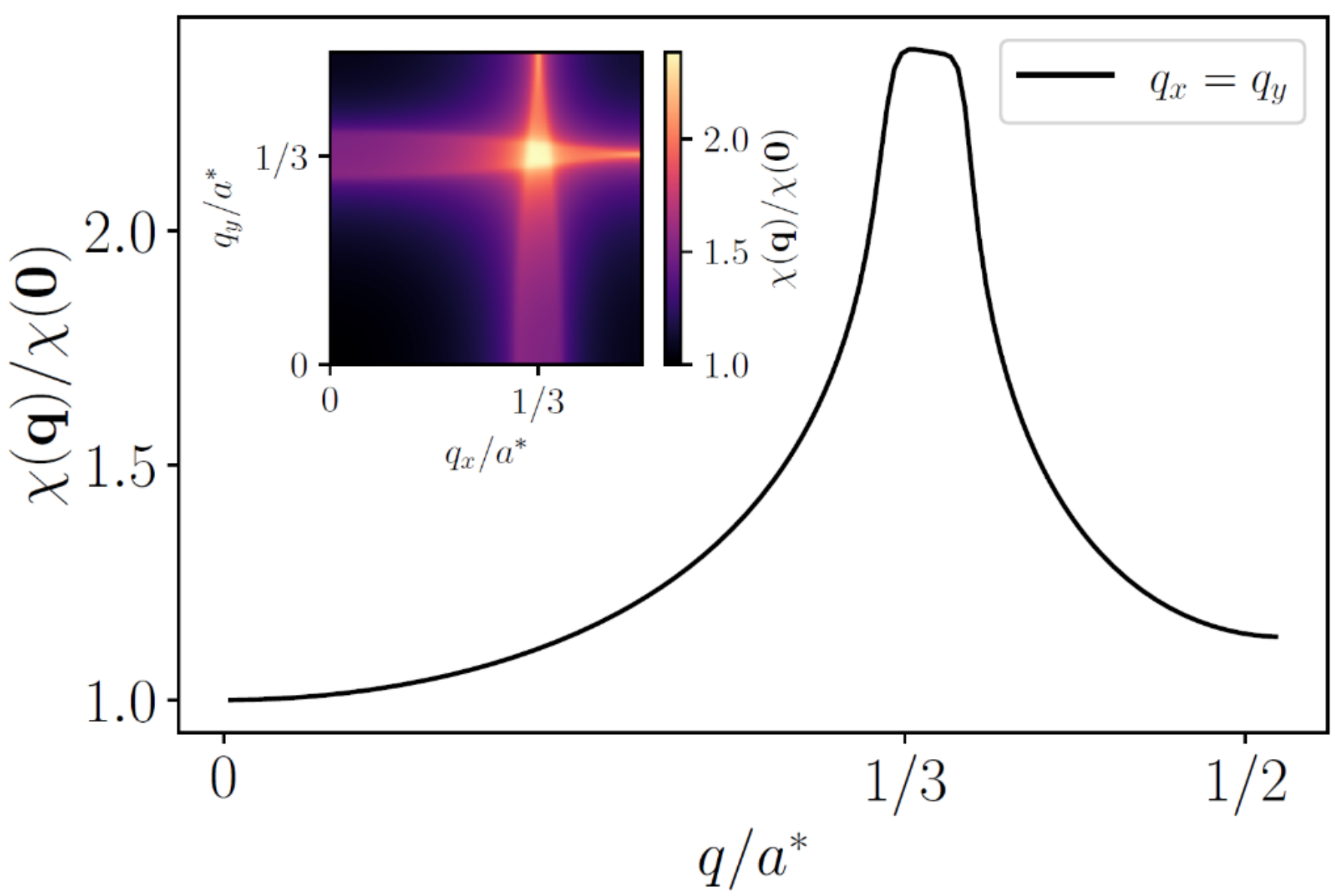

Fig. S14. The susceptibility response of an undistorted square lattice monolayer with the electronic dispersion from the main text. The inset shows the response over a wider section of momentum space, while the main part of the figure shows the cut along the line q_x=q_y.

We observe that the deviation from the commensurate phase is very small and therefore we approximate the ground state with the commensurate situation. At no stage do we enter into the incommensurate phase. To make this point clear, we modified the 5th paragraph in the Discussion section of the main text:

"First, our calculation confirms that for the monolayer, consistent with experiments, the Lindhard response exhibits a peak very close to $q = (1/3a^*, 1/3a^*)$, where a^* is the reciprocal lattice constant of the monolayer unit cell (i.e., rotated by 45º w.r.t. the unit cell of $EuTe_4$ and scaled accordingly50). For simplicity, we approximate the situation with a commensurate wavevector and parameterize the distortion using the parameter α (see Fig. 1b), which is determined self-consistently (see Methods)."

3) Referencing prior work on a switching in a related material 1T-TaS2 with applications in memory would benefit the readers (see "Electrical gating of the charge-density-wave phases in two-dimensional h-BN/1T-TaS2 devices", ACS Nano, 16, 11, 18968 (2022); Appl. Phys. Rev., 10, 041401 (2023)).

Reply: We agree these are relevant references and have included them in the new version of the manuscript.

4) The authors mention Joule heating in several places. Is it possible to provide estimates for the temperature rise due to Joule heating (self-heating) for their devices?

Reply: We estimate that the upper limit for the change in temperature for the 30 ns pulse (shown in Fig. 2c) is only 7 K. The details of the calculation for the change in temperature are presented in the supplemental material (Supplemental Note 5). This is consistent with IV measurements at 300 K that do not indicate a significant change in temperature during the pulse.

We updated the discussion of the Joule heating effect in the Discussion section of the main text with explicit reference to the relevant section of the supplemental material (Supplemental Note 5).

"… For the 30 ns pulses applied at room temperature, we calculate that the total energy deposited in the device is only 13 pJ, which limits the temperature increase during the pulse to 6.6 K (see Supplementary Note 5). …"

Reviewer #4 (Remarks to the Author):

The authors present a study of a layered CDW material, $EuTe_4$, which exhibits several distinct CDW orders, and a hysteretic behavior in the resistivity as a function of temperature. The authors show that by applying voltage pulses they can drive the material into a different point on the resistivity curve, suggesting that this is a hidden state in the material of some sort. They go through some lengths to show that this is likely not a thermal effect, which seems quite reasonable based on their results. They also show that whatever they are seeing occurs rapidly, or at least shorter than they can resolve. The state the system is driven into appears to be stable for long times, and at non-cryogenic temperatures. They report that the system is likely modified by an electric field effect. Some theoretical analysis of the free energy in a restricted range is presented based on the Peierels/nesting model of CDW formation.

Overall, the experimental observations are worthwhile, and demonstrate interesting behavior of this material under new conditions. The theoretical analysis is fine, although I have some questions (below). Mostly, the manuscript does not make the case that this is some new state sufficiently clear. I agree with the authors that it is probably non-thermal, and the change in the resistivity is an indicator that something is different, but the conclusion that this is a non-thermal hidden state needs stronger backup than that. It could, for example, a more disordered version of the existing state, or some other disordered effect that has occurred due to the applied field.

Reply: We thank the reviewer for describing our experiments as "worthwhile" and "demonstrating interesting behavior". While our measurements clearly show that electrically excited states are non-thermal, we agree that further measurements would be needed to resolve the nature of the new order and classify it as hidden. As the reviewer suggests, we have removed the word hidden to describe the new state and now reference it either as "non-equilibrium" or "non-thermal".

On the theory side, the analysis seems oversimplified. There is nothing a priori wrong with it barring the question below, but it seems like this material is exhibiting something new. How is it then that it should be captured by a standard Peierls picture, which in any case should have picked up the thermal phases and others like it?

Reply: This is not a standard Peierls picture, as a crucial effect is the coupling between layers. There is remarkably little literature on how energetics is modified (except for the one-dimensional case, e.g. J.J.M Slot et al., Z. Phys. B - Condensed matter 66, 107-123 (1987)) and our work is the first one to show that interlayer coupling leads to an energy landscape with many metastable minima. We consider this as an important message of the paper and to avoid confusion, we have modified the 5th paragraph in the discussion part of the main text as:

"To theoretically explore the microscopic origin of these free-energy local minima, we extend Peierls' theory of charge-density waves to several coupled layers as relevant for $EuTe_4$."

Moreover, if the energy barrier is really just 15 meV, one would expect thermal fluctuations to appear quite readily, or at least to have some strong temperature dependence on that scale which seems to be absent in the data.

Reply: As we have already emphasized in the main text, this number should be understood as a very crude approximation and a more detailed first-principle calculation is required to provide a more accurate estimation. Nevertheless, 1st order phase transitions would lead to the phase separation of the two phases, namely, different directions of weakly coupled layers due to thermal excitation. Previous STM experiments [Q. Q. Zhang 2023] hint at the coexistence of two polarizations already in the equilibrium low-temperature states, consistent with our expectations. In the coexistence picture, we do not expect any sharp features, but rather a broad hysteresis on the above-mentioned energy scales.

Thus, while I find this work interesting, I do not think it warrants publication in nature communications without a more thorough study of what the actual changes in the material are.

Reply: We greatly appreciate Reviewer #4's recognition that our work is both worthwhile and interesting. We hope that our responses have clarified the earlier misunderstandings and that the revised manuscript now communicates our findings more effectively.

Our results provide robust evidence that the switching mechanism is fast, non-thermal, and driven by an electric field. Beyond the fundamental insights into CDW manipulation and metastability, our demonstration of non-volatile, energy-efficient resistance switching highlights clear potential for memristor applications.

Equally important, our study opens up compelling future directions: the precise nature of metastable CDW states in $EuTe_4$ remains unresolved, and addressing this will likely require extensive future experiments, e.g., diffraction. We believe that these open questions will be of immediate interest to a broad scientific community.

Minor points:

Do you have a sense as to how big the electric field is? Is it a sensible scale to cause the electrical switching in a CDW?

Reply: We thank the reviewer for this comment, as the values of an electric field are indeed relevant for the switching behavior. Calculating the threshold electric field at room temperature from both devices, we obtain the threshold fields ED1 = 0.86 kV/cm, and ED2 = 0.75 kV/cm. Both devices therefore demonstrate similar switching behavior. This is comparable to the depinning field in 2D CDW materials, such as 1T-TaS2, which has the CDW depinning field of 1-5 kV/cm (Ref. 47). Therefore, the scale of the electric field observed here is sufficient for inducing a change in the CDW order.

We have extended the discussion section of the main paper (2nd paragraph) with the calculation of the switching electric field:

“.. Calculating the switching electric field at room temperature from the device dimensions and switching voltage, we obtain the threshold values of ED1 ~ 0.86 kV/cm, and ED2 ~ 0.75 kV/cm (see Supplementary Notes 8 and 9 for details). .. ”

The details of threshold electric field calculation are presented in Supplementary Notes 8 and 9.

Fig 1 -- it is generally unclear which aspects of the figure are being referred to in the main text. For example, where is the jump in resistance due to the 2.7V pulse?

Reply: A jump in resistance in Fig. 1e is indicated with the white arrow. To improve clarity, we expanded the relevant first paragraph of the Results section of the main text:

“.. We observe that by applying a current pulse to the cooling branch at 300 K, the resistance of the device increases and is stable on the laboratory timescales. By increasing the pulse magnitude up to 2.7 V, a large change in device resistance can be induced (as indicated with a white arrow in Fig. 1e). ..”

Additionally, the figure caption has been expanded to reference the jump in resistance.

“.. Thermal erase cycle (blue) following the resistance switching by a 2.7 V pulse at 300 K (indicated with a white arrow). ..”

The discussion in comparison to the semiconductor is not very clear. I gather that the goal is to say that the switching is non-thermal because a semiconductor (which this ostensibly is based on the IV curve) shows sublinear behavior in the IV curve and decide D1 does not. Currently, it reads as if a comparison is made to say that D2 is not a semiconductor.

Reply: Given that $EuTe_4$ is a semiconducting material, our intention was indeed to distinguish the observed switching behavior from that attributable to joule heating in a typical semiconductor.

To improve clarity, we have expanded the Figure 2 caption:

“.. The inset shows a schematic of a sub-linear I-V response of a semiconductor with a significant change in temperature due to Joule heating. A distinct response of a semiconducting $EuTe_4$ device indicates minimal change in temperature during pulse application. …”

We also made an addition to the main text:

“.. This contrasts with Joule heating response of a semiconductor which would result in a sub-linear I-V curve as schematically shown in the inset to Fig. 2b. ..”

These revisions now more explicitly explain our comparison of the experimental I-V characteristics to the expected response from semiconductor heating, emphasizing our argument for a non-thermal switching mechanism.

Finally, a figure of the Lindhard function at w=0 would be very helpful. How distinct is the peak? Rare-earth tritellurides typically do not show a sharp peak in the susceptibility (see e.g. Johannes and Marin, PRB2008). Fig 4 is not helpful because it has no proper scale.

Reply: We provide a figure of the Lindhard response for the monolayer tight-binding model, where the inset shows the data over the full q-space, while a slice along the diagonal is shown as the main curve. The peak is at the expected momenta, although similar to the discussion in PHYSICAL REVIEW B 77,

165135 2008, its value is reduced to relatively weak enhancements. We should comment that our work provides a more direct calculation, namely the comparison of the free energy of the state with and without the broken symmetry and the former is substantially lower. Such a calculation includes the effect of the momentum-dependent electron-phonon coupling, see also reply to Reviewer #5. In our view, this is a more direct indication of the CDW formation. The data shown is for \beta=40 (i.e., room temperature).

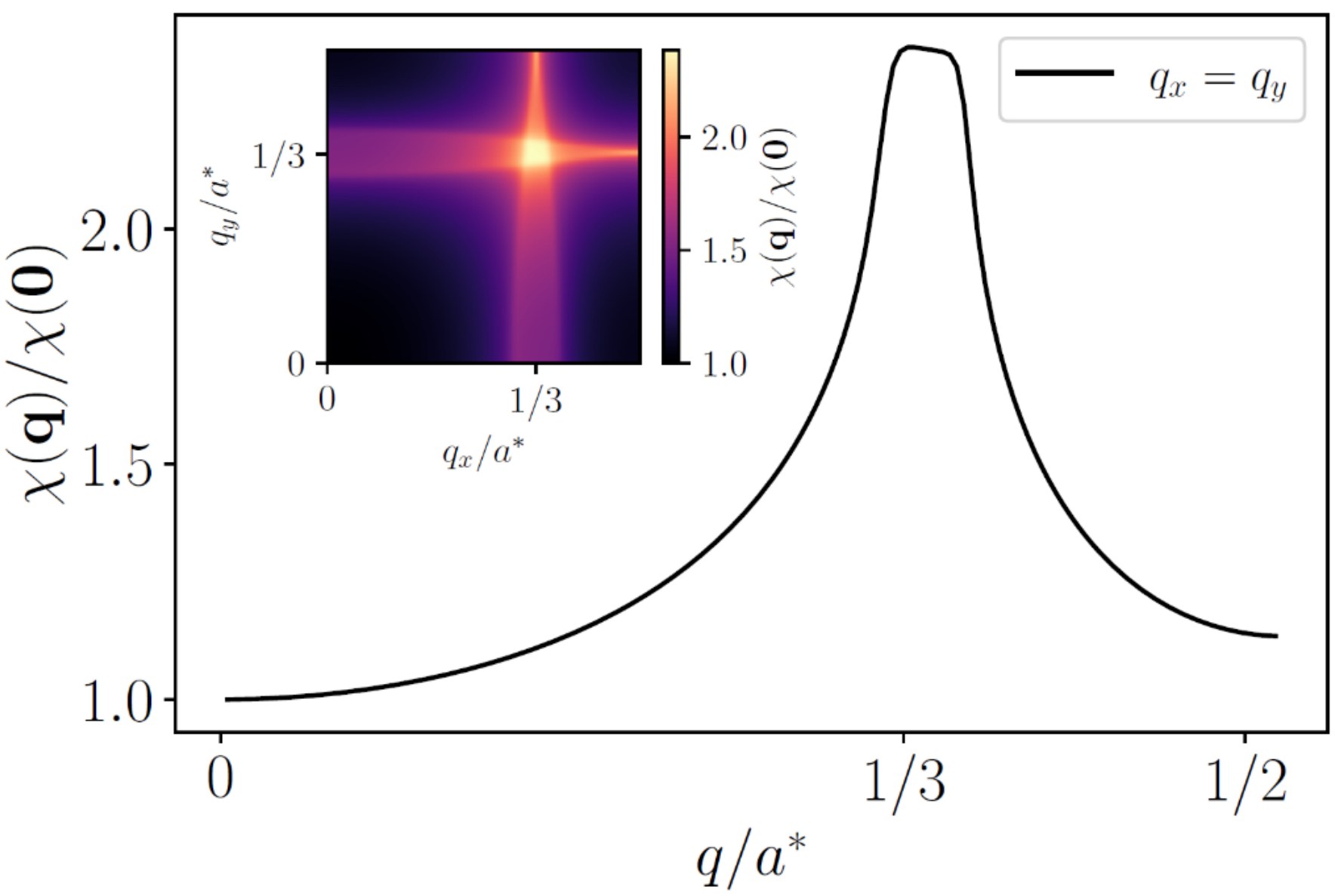

Fig. S14. The susceptibility response of an undistorted square lattice monolayer with the electronic dispersion from the main text. The inset shows the response over a wider section of momentum space, while the main part of the figure shows the cut along the line q_x=q_y.

In accordance with reviewer comments, we updated the colorbar scale of the zoomed-in sections of the bilayer Lindhard response, highlighting that the split peaks maintain a significant albeit slightly lower magnitude. We have added the Lindhard function in the Supplementary Information (Supplementary Note 12, Fig. S14).

The expression for the Lindhard response is odd. It appears that m,n index the bands and I,\alpha index the orbitals. Without the orbital sum, this is an orbitally resolved susceptibility. Why take the sum of all these elements? A measurable susceptibility is the trace. Please confirm that this is the appropriate susceptibility.

Reply: As the reviewer indicated, m and n do index the bands in the diagonal basis. The indices i and \alpha, however, originate from the trace (i) and matrix multiplication (\alpha).

Starting from this expression for the susceptibility involving matrix-valued Green's functions:

$$\chi^R(q,t) = -\frac{i}{N}\theta(t)\sum_{k} \mathrm{Tr}\left[\boldsymbol{G}^{<}_{k+q}(t)\boldsymbol{G}^{>}_{k}(-t) - \boldsymbol{G}^{>}_{k+q}(t)\boldsymbol{G}^{<}_{k}(-t)\right]$$

we rewrite it in terms of matrix elements, converting the trace into a sum over i and the matrix multiplication into a sum over \alpha i.e.,

$$\chi^R(q,t) = -\frac{i}{N}\theta(t)\sum_k\sum_{i,\alpha} G^<_{k+q,i\alpha}(t)G^>_{k,\alpha i}(-t) - G^>_{k+q,i\alpha}(t)G^<_{k,\alpha i}(-t)$$

To highlight the familiar "difference of occupation divided by difference of energy" form, we then express the greater/lesser Green's functions in terms of the diagonal basis (sum over m, n) and FT to frequency space, leaving us with the overlap integral factor (sum over i, \alpha) as written in the submitted text.

$$\chi^R(q,\omega) = \frac{1}{N}\sum_k\sum_{m,n}\frac{F_{k+q,m}-F_{k,n}}{\omega+i\eta+D_{k+q,m}-D_{k,n}}\sum_{i\alpha} U_{k+q,im}U^*_{k+q,\alpha m}U_{k,\alpha n}U^*_{k,in}$$

where we used the following definition of the lesser and greater Green's function for translationally invariant systems:

$$G^<_{k,ij}(t) = i\sum_\alpha U_{k,i\alpha}e^{iD_{k,\alpha}t}F_{k,\alpha}U^*_{k,j\alpha}$$
$$G^>_{k,ij}(t) = -i\sum_\alpha U_{k,i\alpha}e^{iD_{k,\alpha}t}\left(1-F_{k,\alpha}\right)U^*_{k,j\alpha}$$

The above expression for the Lindhard response can also be written as

$$\chi^R(q,\omega) = \frac{1}{N}\sum_k\sum_{m,n}\frac{F_{k+q,m}-F_{k,n}}{\omega+i\eta+D_{k+q,m}-D_{k,n}}\left\|\left\{\boldsymbol{U}^\dagger(k+q)\boldsymbol{U}(k)\right\}_{mn}\right\|^2$$

where the confusion about the summation indices is now compressed into the magnitude of the product of eigenvector matrices. We thus thank the reviewer for pointing out this issue and have modified the equations in the Methods section accordingly, so they align with the more commonly accepted form.

Reviewer #5 (Remarks to the Author):

In this work, Venturini and colleagues studied the electrical resistivity switching phenomena in a recently-discovered CDW material, $EuTe_4$. The team found that the material can enter a metastable, higher-than-normal resistivity state following the application of electrical pulses, which is very stable if left unperturbed, but can return to the equilibrium state after thermal cycling. The authors presented convincing evidence that the electrical pulse-induced switching cannot be explained by a trivial joule heating scenario, and the switching occurs shorter than 30 ns. Quite remarkably, the switching is

somewhat deterministic for both ways depending on the amplitude of the current pulse, as shown in Figure S6.

Overall, I find the results very interesting even though there has been an earlier demonstration of a similar phenomenon using ultrafast laser pulses in Ref. [39]. From an application standpoint, electrical switching at room temperature is much simpler and hence more appealing than the complex setup of an ultrafast laser. The author team also conducted a series of systematic experiments that aimed to constrain the possible mechanisms of this switching phenomenon, which, despite a lack of definite conclusions of the microscopic switching pathways, are still very valuable. However, I have a number of technical concerns, which may prevent readers from fully appreciating the significance of these results. I hence recommend a revised manuscript to be published in Nature Communications after the authors address the following points.

Reply: We are very pleased with the overall positive assessment of our work by the reviewer, and we certainly share their view that the deterministic behavior of switching is remarkable. We wish to thank the reviewer for their detailed reading and comments, which certainly helped us to improve our work. We are happy to address their concerns in a point-by-point response below.

1. Although I am convinced about the non-thermal nature of the switching as explained in the first paragraph of the “Discussion” section on page 7, I have some reservation about the estimated 6.6 K (which is quoted as 7 K in the main text) temperature increase, which is calculated on page 5 of the Supplementary Information. In this 6.6 K estimate, the authors assume that the 13 pJ energy is uniformly distributed throughout the sample volume between the electrodes. However, for semiconductors, spatial distribution of current flow is rarely uniform, and in the extreme case, there can be a filamentary current flow. I would expect this non-uniformity to hold especially for $EuTe_4$ if there are different metastable domains. Is there a way to show that the current flow is indeed uniform as the authors assumed? Or is there an alternative way to estimate an upper bound on the temperature increase of the region of the sample that switched, which is not necessarily the entire sample volume between the two electrodes?

Reply: We appreciate the reviewer's question regarding the assumption of uniform current distribution. While filamentary switching is common in some emerging memory devices (e.g., 1T-TaS_2 where a low-resistance channel forms), our observations are not consistent with such behavior in $EuTe_4$ devices.

For filamentary switching, a narrow low-resistance channel appears in a device. In such cases, the appearance of a low resistance channel drives a large current through the filamentary path, causing localized heating. Crucially, in $EuTe_4$ devices, the resistance increases during the switching.

If the switching were confined to a narrow filament transitioning to a high-resistance state, its impact on the device's total conductance would be minimal, as the current would continue to flow through the surrounding low-resistance part. The observed large increase in total device resistance is therefore inconsistent with a filamentary process and indicates that a majority of the device volume participates in the transition.

A strongly non-uniform or filamentary current flow would lead to significant localized Joule heating. Given the semiconducting nature of $EuTe_4$, this would cause a substantial reduction in the device

resistance during the pulse. This would be directly observable in our pulsed I-V measurements as a sub-linear I-V response, which is inconsistent with our measurements. The absence of this sub-linear response provides strong evidence that significant localized heating does not occur, which in turn suggests that the current flow and subsequent switching are reasonably uniform across the device.

We added a comment to the discussion in the main text to explicitly state that our data is not consistent with the filamentary switching process:

"...Unlike 1T-TaS2, where switching in a device appears along a narrow channel[Ref. 43], the overall large increase in resistance in $EuTe_4$ devices indicates that the majority of the device is involved in the switching process (see Supplemental Note 5 for further details). .. "

Supplementary Note 5 has been expanded with the discussion of potential non-uniformity of the current flow and switching volume. We have also changed the value of the change in temperature in the main text from 7 K to 6.6 K for consistency with the supplementary section.

2. Still on the point of joule heating, I find it surprising when comparing Figure 2c and Figure S6. In Figure 2c, the pulse length is varied by 8 orders of magnitude at a constant voltage pulse amplitude, meaning that the energy carried by the pulse also changed by 8 orders of magnitude. However, that has no effect on the final, electrically switched state. The authors interpreted this observation as evidence for a non-thermal switching behavior. In Figure S6, by changing the pulse amplitude from 300 µA to 480 µA while keeping the pulse duration the same at 200 µs, they can switch between a nonthermal "writing" pulse and a joule-heating "erase" pulse. This looks very strange: applying a 2.0 V pulse for as long as 1 second in Figure 2c still has no joule heating effect (estimated current is 2.0 V / 3200 Ω = 625 µA), but applying a 480 µA current for 200 µs in Figure S6 enters the joule heating regime.

Reply: Thermal erase with electrical pulses is experimentally challenging and requires a certain level of fine-tuning for reliable operation. We noticed that without an optimized procedure, a large and rapid change in temperature due to a high-voltage pulse can easily damage the device. As electrical pulse erase is not a key part of this work, we wanted to minimize the risk of device destruction and use moderate pulses for the proof-of-concept for the pulse erase.

The key difference between pulses in Fig. 2c and erase pulses in S6 is that pulses in S6 were applied at a much higher temperature of 400 K. At higher temperatures, we expect that a smaller temperature change would lead to relaxation of metastable states. Therefore only a modest amount of additional Joule heating from the electrical pulse (480 µA for 200 µs) is needed to observe relaxation of state (reduction of resistance). This is consistent with pulsed IV measurements performed at 400 K (Fig. 3c), where device resistance increases due to electric field switching up until around 400 µA. For larger current values, the device resistance starts to decrease, due to the Joule heating and increase in temperature of the device.

We expanded Supplementary Note 6 with discussion of the role of temperature in electrical pulse erase.

3. The authors concluded from Figure 3e that "the electric field drives the switching to the novel states and not the current injection". I did not quite follow the physical reasoning here. Why does a constant voltage threshold in Figure 3e indicate that it is the electric field that drives the switching behavior? And can the author relate the voltage value to a threshold electric field, and discuss how the field strength relates to known microscopic parameters of $EuTe_4$?

Reply: We agree that the reasoning for attributing the switching to the electric field deserves a more detailed explanation.

The switching behavior looks very similar across the explored temperature range, with a gradual increase in resistance above a certain current/voltage value (see Figs. 3c and 3d). This indicates the same underlying mechanism is responsible for switching at all temperatures. If switching were driven by charge injection (current density), one would expect the threshold current to be the parameter that remains relatively constant across different temperatures. Conversely, if driven by the electric field, the threshold voltage should be constant. Our data in Fig. 3e clearly show the latter: the threshold current varies by almost two orders of magnitude, while the threshold voltage remains remarkably stable. This suggests the switching is driven by voltage or electric field instead of current. Analogous argument was used to understand switching in 1T-TaS2 as being driven by charge injection (Vaskivskyi et al., Nat. Comm. 2016).

The last paragraph in the results section of the main paper now reads:

".. As shown in Fig. 3e, the threshold current for switching is significantly reduced as the temperature is lowered, while the threshold voltage remains at a similar value throughout the explored temperature range. A mechanism primarily driven by charge injection would be expected to occur at a constant threshold current, as has been observed in 1T-TaS_2 [Vaskivskyi et al., Nat. Comm. 2016]. In contrast, our observation of a constant threshold voltage strongly indicates that the electric field is the primary driver of the switching."

4. The authors mentioned the recent experiments in Ref. [39] about the laser-induced non-volatile switching in $EuTe_4$. I think the readers will benefit from a more thorough discussion about the similarities and differences between the phenomenology of the present electrical-switching experiments and the previous laser-switching experiments.

Reply: We agree with the reviewer that a more thorough comparison with optical switching is worthwhile and would benefit the readers. We expanded the comparison to optical switching in paragraph #3 in the discussion section of the main text that now reads:

".. Our electrical switching results show several parallels with recent optical manipulation experiments in $EuTe_4$ done at room temperature [Liu et al., Nat. Comm. 2024]. This indicates that non-volatile CDW switching is intrinsically linked to the unique CDW physics of $EuTe_4$. On the cooling branch of the thermal hysteresis, both electrical pulses and optical excitation increase the resistance. On the heating branch, the response is similarly non-monotonic: lower intensity stimuli (weaker electric fields or lower laser fluences) decrease the resistance, while higher intensity stimuli cause it to increase again. Furthermore, much like high-fluence optical experiments, strong electrical pulses can induce a non-thermal state far outside the equilibrium hysteresis loop. In contrast to optical switching, only low voltage is required for device operation, allowing us to clearly isolate the non-thermal pathway and demonstrate energy-efficient memristive control. …"

5. Comparing the equilibrium R-T curves between Figure 3a and Figure 1d, besides the one-order-of-magnitude difference in the actual resistance (presumably due to sample size and contact resistance), the two hysteresis loops also look quite different. Can the authors comment on which phenomenology regarding the resistivity switching is sample dependent, and which is not? For instance, from Figure 3e, is

the threshold electric field (need to convert voltage to field) at each temperature sample dependent? And is the percentage resistance increase under the same electric field the same across samples at a fixed temperature?

Reply: It is correct that there are some differences between devices. The difference in resistance between the two devices is perhaps a bit smaller than it seems: at room temperature on the cooling branch, the resistance of device D1 is around 5.5 kohm, while the resistance of D2 is around 1.5 kohm. The reviewer is correct that the dimensions of devices are different, with device D2 being substantially thicker, explaining lower resistance of device D2 compared to D1. We have now added all the device dimensions to the Methods section of the manuscript.

While all our devices exhibit semiconducting behavior with a wide hysteresis loop, there is indeed a certain variation in the shape of the hysteresis loop. Variation of the hysteresis curve shape of bulk $EuTe_4$ crystals can also be observed by comparing different works across the literature [e.g., (Lv et al., PRL 128 2022), (Zhang et al., PRB 107 2023), (Takahashi et al., PRX Energy 4 2025)]. In the recent work, authors speculated that a variation of $EuTe_4$ hysteresis could be due to impurities of the Eu source [Takahashi et al., PRX Energy 4 2025]. Additionally, CDW materials are sensitive to strain, which could be present to a small extent in fabricated devices. Given the known variability of $EuTe_4$ hysteresis shape, we speculate that differences in device thickness, local strain due to lithography fabrication, or a small amount of crystalline defects could explain the observed level of device variability. Despite these moderate variations, we wish to emphasize that the key phenomena for our discovery are robust and consistent across devices. All devices exhibit hysteretic semiconducting behavior and the same response to electrical pulses: a non-volatile increase in resistance (by starting from the heating branch) that is triggered already at low electric fields.

For clarity, we expanded the paragraph in the results section with the following sentence:

"... We note that the hysteresis curve of D2 (Fig. 3a) exhibits some variation in shape compared to device D1. Such variability is also observed in the literature, where the exact shape of the hysteresis appears to differ between crystals used in separate works [32,38,41] and could be due to a small level of impurities of the Eu source[41], difference in device thickness, or local strain. .."

Comparing the threshold electric field at room temperature for devices D1 and D2, we obtain the threshold fields ED1 = 0.86 kV/cm, and ED2 = 0.75 kV/cm. Again, this demonstrates consistent behavior across both devices.

We have updated the main text with these values in the Discussion section:

"... Calculating the switching electric field at room temperature from the device dimensions and switching voltage, we obtain the threshold values of ED1 ~ 0.86 kV/cm, and ED2 ~ 0.75 kV/cm (see Supplementary Notes 8 and 9 for details)."

Supplemental Notes 8 and 9 were expanded with calculations of the threshold electric fields for both devices.

6. In the introduction paragraph on page 1, the authors mentioned "As [resistance switching in CDWs] is based on changes to the electronic instead of the structural order, such switching was shown to be ultrafast, ultraefficient, and has high endurance." I am confused by this comment because doesn't the

periodic lattice distortion of the CDW (for both 1T-TaS_2 and $EuTe_4$) change when it enters a metastable state? For 1T-TaS_2, the authors mentioned that there is a "network of CDW domains and domain walls", and the atomic positions at those domains/domain walls are presumably modified in addition to electron density modification. Similarly, for $EuTe_4$, the authors mentioned the out-of-plane arrangement of bilayers and monolayers of Te, and I would imagine that the Te ions have to move when going from the equilibrium state to a metastable state. In that case, the structural order is also involved.

Reply: We thank the reviewer for raising this point. We agree that a change in the CDW state inherently involves some movement of atomic positions, as the atoms accommodate the new charge order, and our original phrasing was imprecise. The reason for this sentence is to contrast the CDW switching to the switching mechanism of phase change memory devices (e.g., melt-quenching between amorphous and crystalline phases). There, switching is slower and requires a significant energy investment to drive the material across a structural phase transition via heating.

We corrected the relevant sentence in the introduction section of the main text, which now reads:

"As the switching is driven by electron reconfiguration, such a mechanism was shown to be ultrafast, ultraefficient, and has high endurance."

7. The two-way resistance switch in Figure S6 is quite subtle, where the change in resistivity is very small for a semiconductor. Have the authors found a similar two-way resistance switching at cryogenic temperatures? Or is there a reason that the low-temperature switching is not two-way?

Reply: As discussed in reply to question #2, we find that at higher temperatures, the state can be erased even by modest current pulses (moderate heating). Without optimization of the erase procedure, the device can be damaged with more intense heating pulses, which is why we opted for a modest erase at a higher temperature to demonstrate the feasibility of an all-electronic erase. Indeed, optimizing the electrical erase procedure at different temperatures could be an interesting avenue for further application-oriented research.

8. I find the theory discussion on page 7 to page 9 can benefit from a more rigorous exposition. I have a few comments:

(i) The adoption of Peierls' description of the CDW in $EuTe_4$ is rather simplistic and possibly wrong. The authors assumed a momentum-independent electron-phonon coupling g on page 12. For a closely related CDW material, RTe3, it has in fact been shown that anisotropic electron-phonon coupling, where g is both momentum and phonon-branch dependent, is the dominant factor; see Frank Weber's inelastic X-ray scattering results in Phys. Rev. B 91, 235146 (2015).

We would like to point out that the electron-phonon coupling we adopt is, in fact, momentum dependent. To avoid further confusion, we rewrote the corresponding equations in a form more consistent with literature convention, highlighting this momentum dependence. Consistent with the reference mentioned by the referee, the electron-phonon interaction g(k,q) is peaked at the momentum $q=q_{CDW}$. We have modified methods such that this is clear.

(ii) The bigger question about the theory discussion is that it does not seem to show how and why the application of electrical pulses can switch the systems between different metastable states. The

presented theory only shows the possibility for metastable states to exist, which is already known from previous experimental studies cited by the authors. I think what is missing here is

(a) the connection between the theoretically proposed metastable states to the different resistivities associated with these states,

Reply: This is an excellent question. We have checked the dispersion of both states in Fig. 4 and observed that the bandgap in the two phases is different. In the phase marked with blue color ($\alpha > 0$), the gap is consistently larger than in the state marked with the red color ($\alpha < 0$), which also means that the resistivity is larger in the former case, see S13. In the concrete calculations, the bandgap of the former is 564 meV and the latter 520 meV. The typical switching starts from the cooling branch of the hysteresis with lower resistivity (equivalent to the red state <0) and switches to a higher resistive state (equivalent to the blue state >0). We comment on this observation in the main text:

".. A comparison of the band structures for the two states shows that the state with $\alpha > 0$ always exhibits a larger band gap than the state with $\alpha < 0$ (see Fig. S13). Consequently, the resistivity of the former is also expected to be higher, which would be consistent with experimental observations. .."

(b) what happens microscopically when an electrical pulse passes through the sample, and

Reply: The nonequilibrium dynamics in the model proposed within this work is indeed an important next step and we have already taken steps along these lines. It turns out that the E-field response is rather complex and we would prefer to provide a detailed analysis as a follow-up to avoid misunderstandings due to a "quick-and-dirty" presentation.

(c) how to reconcile different features of the switching phenomenology in Figures 1 to 3, such as it is electric field driven, it increases instead of decreasing the resistivity, and why the electrically-induced metastable states are different from the metastable states in the hysteresis loop.

Reply: We answered the question of " increases instead of decreasing the resistivity" above. Switching phenomenology and why the metastable state is different requires real-time simulation in the future. We speculate that the former leads to coexistence of both phases with rations that are not determined by the thermal fluctuations

(iii) As a minor note, it would be beneficial to draw the coordinate system when the authors switched to the q = (1/3a, 1/3a) notation on page 7 even though the in-plane CDW is along the b axis, as mentioned on page 2. I am afraid not all readers may catch the 45° rotation between the square net of the Te monolayer and the actual unit cell.

Reply: We added a discussion of the unit cells in the Supplementary Note 11 with a new Figure S12 as well as the reference V. Brouet, Phys. Rev. B 77, 235104 (2008), which illustrates the question of the coordinate system. We added a mark in the discussion part of the main text:

".. the Lindhard response exhibits a peak very close to q = (1/3a*, 1/3a*), where a* is the reciprocal lattice constant of the monolayer unit cell (i.e., rotated by 45º w. r. t. the unit cell of $EuTe_4$ and scaled accordingly), see S12. .."

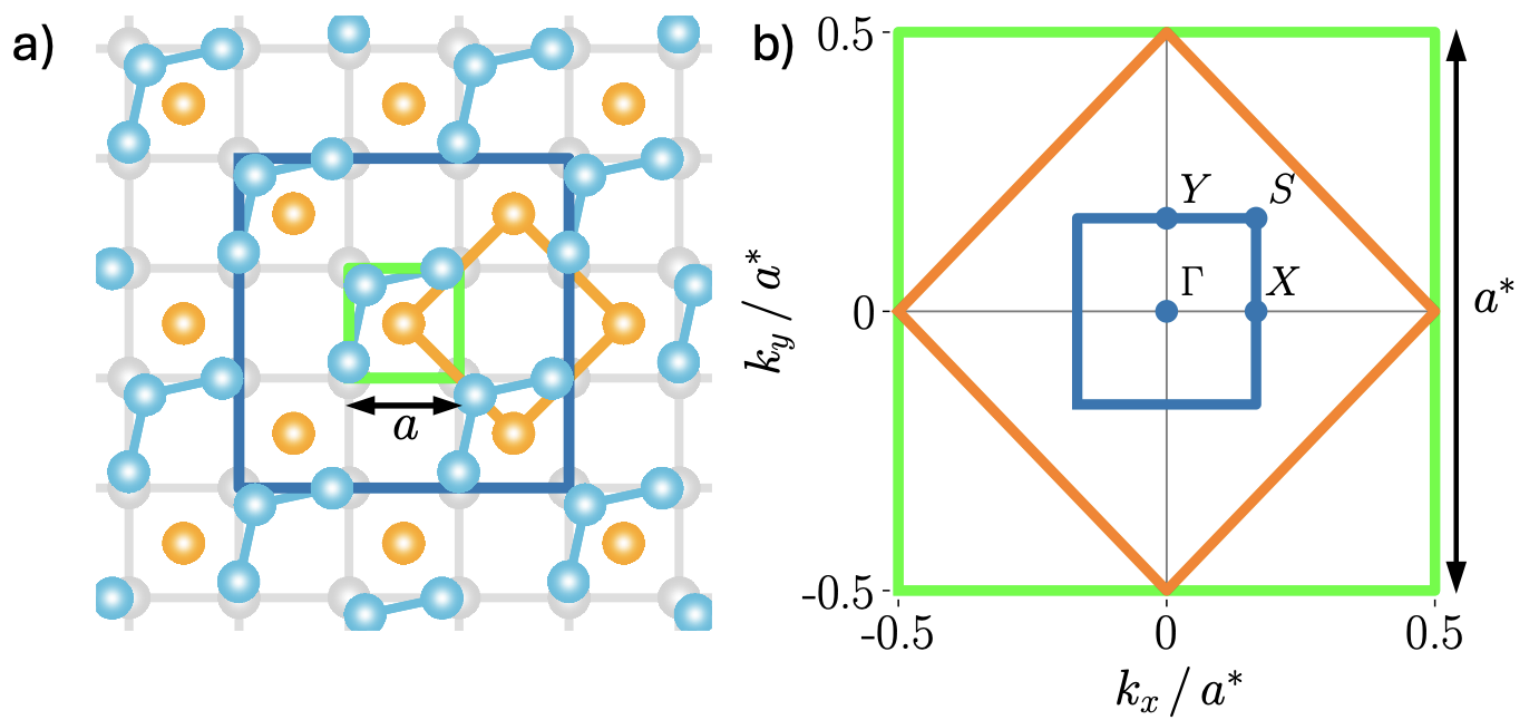

Fig. S14. a) The real-space representation of a pure Te sheet (gray – undistorted, blue – distorted) with Eu atoms from the corrugated Eu-Te layer below shown in orange. The undistorted Te sheet unit cell is shown in green, and the unit cell of the in-plane CDW phase is shown in blue. The in-plane unit cell of $EuTe_4$ is shown in orange. b) The first Brillouin zones corresponding to the real-space unit cells from (a) are shown using the same colors as in (a). The high-symmetry points of the 2D reduced Brillouin zone are labelled as well.

Reviewer #1 (Remarks to the Author):

For our previous queries, the authors have provided detailed clarifications and arguments supported by additional experimental data and theoretical analysis.

The revised version appears more in-depth and rigorous. In general, again, this work demonstrates the non-thermal and nonvolatile memristive switching at room temperature in CDW EuTe4 induced by pulsed electric fields, which exhibits unique kinetic characteristics distinct from those of previously reported systems. Given the complex nature of the intrinsic interactions in this material, we agree that a complete understanding of the underlying dynamics will require further experimental investigation beyond the scope of a single article.

We have no further concerns regarding the revised manuscript and recommend its acceptance for publication.

Reply: We thank the reviewer for the positive evaluation and for recommending our work for publication.

Reviewer #3 (Remarks to the Author):

The authors responded properly to reviewers' questions and requests. I recommend the acceptance of the revised version.

Reply: We thank the reviewer for recommending our work for publication in its current form.

Reviewer #4 (Remarks to the Author):

I will defer to the other reviewers on the experimental aspects. I do agree with Referee #1 that an absence of theory-experiment correlations is regrettable, and that such an analysis would much improve the manuscript. In my estimation, this remains necessary before publication in Nature Communications, although the other reviewers disagree with me on this.

The authors have improved the description of the theoretical work performed, but it is still unclear. The issues below should be addressed and corrected before publication is warranted.

Reply: We thank the reviewer for their comments and appreciate their detailed analysis of our work. We share the reviewer's opinion that a more thorough connection of theory and experiment is desirable. However, we again point out that the development of a more involved theoretical framework would be better suited for a separate publication.

* There could be more clarity on the distinction between orbital and band indices in the manuscript. I gather that the two orbitals do not interact at all, but this is not clear.

Reply: The reviewer has correctly concluded that the p_x and p_y orbital spaces are indeed decoupled. To improve clarity, we rewrote the relevant parts of the Methods section and adapted the indexing of variables to be consistent throughout the various subsections of the text.

* If the former is true, then why is there a matrix nature to the Hamiltonian? Is this just interlayer coupling, and the authors are considering two independent sets of bands?

Reply: The matrix nature of the Hamiltonian can be considered to originate from two distinct sources:

i) interlayer coupling: We denote these with the variable {x, y} throughout the Methods section. We highlight this in the newly adapted "Lindhard response" subsection of the Methods section. There, we show how the Hamiltonian matrix of a bilayer system without CDW order looks like. As the orbital sectors are decoupled, all matrix elements coupling p_x and p_y electronic degrees of freedom are zero - each orbital space can be treated independently. However, there are non-zero matrix elements coupling states in the same orbital subspace, leading to out-of-plane physics.

ii) CDW order: the breaking of translational symmetry as a result of CDW order leads to backfolding to a reduced Brillouin zone, which in turn leads to the 3x3 Hamiltonian matrix (for a given orbital subspace for a single layer) shown in the "Lattice distortion and Peierls coupling" subsection.

* Reference is made to the Hamiltonian matrix h(k) on line 353, which is not defined, and its connection to the eigenvalues \varepsilon_m(k) -- a variable that also appears on line 330 -- is not clear. If h(k) is intended to mean the one that appears on line 375, then the ordering is incorrect.

Reply: We thank the reviewer for highlighting that the section on the Lindhard response lacks clarity. We replaced the somewhat generic description with one more specific to the problem at hand and amended our explanation. We hope that this aids towards understanding and the ease of reproducibility of the results.

* Line 346 appears like it is missing text.

Reply: We thank the reviewer for pointing this out. The text was missing estimates on the interlayer coupling parameters, which were now added.

* Please clarify what the "canonical displacement variables" (line 379) mean.

Reply: The canonical displacement variables u_q are the normal modes of distortion with non-zero Fourier coefficients of the real-space periodic lattice site displacements. Clarification has been added to the manuscript.

* The band structure in Fig S13 is quite strange. There are discontinuities just to the side of the high symmetry points of the BZ (not right at them); see e.g. the topmost red line crossing the X point or S point as an example. Moreover, if the reduced zone picture is correct, then the bands should approach the Brillouin zone edge with 0 slope. They clearly do not, which suggests they should be plotted in the larger zone.

Reply: We thank the reviewer for pointing out the issues with our presentation of the electronic band structure. We resolved the discontinuity problem by more careful indexing of the k-space path. Motivated by the comments of both Reviewer #4 and #5, we decided to adapt our band structure presentation to ensure easier comparison to experimental results.

With reference to Fig. 1 below, in our work, we focus primarily on the physics of the pure Te sheets (green BZ), and their symmetry broken CDW phase (blue BZ). However, for the purpose of comparison with ARPES experiments, we now plot electronic dispersions along a high-symmetry path related to the

BZ of the undistorted $EuTe_4$ crystal taking into account the presence of the corrugated Eu-Te slabs (orange BZ). Therefore, in the plots of the electronic dispersion, we backfold the bands in the "corners" of the green BZ back into the orange BZ via translation by a wave vector equivalent to the side of the orange BZ.

Our 2D description lacks matrix elements originating from the full 3D structure, and therefore, the backfolded bands do not "interact with the original" bands. There inclusions would lead to a small gap and the velocity at the Brillouin zone edge with 0 slope. The omission of these matrix elements is justified by experimental results, as they are relatively small in value and occur away from the Fermi surface. We hope these changes aid in the understanding of our theoretical results and facilitate comparison to the experiment.

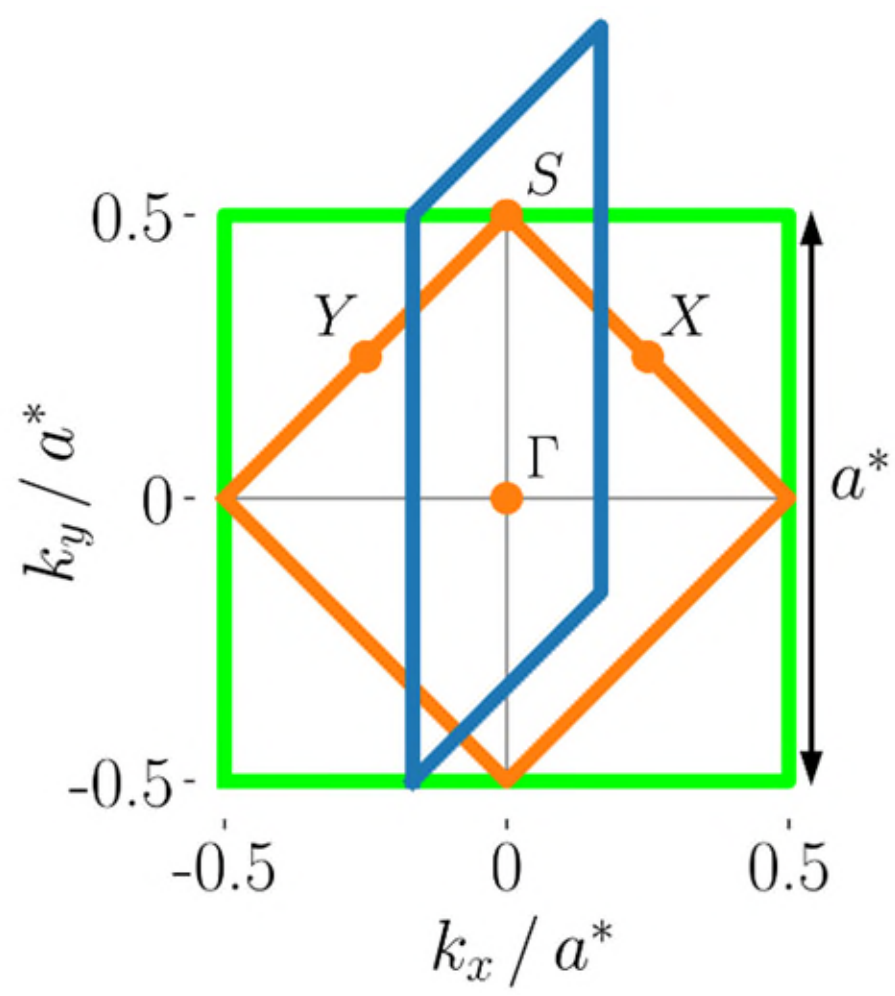

Fig. 1: The three relevant Brillouin zones for our discussion. Green: first Brillouin zone (FBZ) of the undistorted Te square lattice. Orange: FBZ of the undistorted Te square lattice, taking into account the presence of the corrugated Eu-Te slabs (this is the BZ used as a reference to ARPES experiments). Blue: a possible choice of the reduced BZ for the Te sheet following the breaking of the translational symmetry due to CDW order.

* The Lindhard susceptibility (S14) does have a peak, but it is only marginally larger than the background. A momentum-dependent electron-phonon coupling does help to form a clear CDW with a well-defined peak, but I cannot tell from the formalism as outlined where that goes into the calculation. The vertex appears to have an orbital (band?) dependence, but the susceptibility expression does not. If the momentum (orbital/band) dependent vertex simply goes into the mean field CDW procedure that this is fine if clearly stated; it would then also be helpful have a Lindhart susceptibility that incorporates it to be consistent in what is being used for the condensation.

Reply: The bare Lindhard response which we compute only contains information on the electronic degrees of freedom, as opposed to a dressed Lindhard function, which would include the electron-phonon vertex, and which we do not evaluate. The peaks in the bare Lindhard response (a consequence of Fermi surface nesting) are then used as the motivation for the CDW wave vector and the parametrization of the periodic lattice distortion. Our main claim is that the free energy of the system is lowered when entering the symmetry-broken phase with respect to the undistorted state, and this is a much stronger argument than the height of a peak in susceptibility. We have reformulated the method

part such that the inclusion of the momentum-dependent el-ph interaction is as explicit as possible. The susceptibility measures the response of the full density on periodic modulation, and not the response of each orbital, which is why we trace over the orbital degrees of freedom.

Reviewer #5 (Remarks to the Author):

I thank the authors for the revised manuscript and the point-by-point response to my and other referees' comments. While I stand by my previous assessment that the work would be valuable for publication in Nature Communications, there are still outstanding technical points that require additional attention. I list them below.

Reply: We thank the reviewer for careful reading and helping us resolve the technical details of the manuscript.

Concerning my Comment 3 in the previous round of review, I asked, "can the author relate the voltage value to a threshold electric field, and discuss how the field strength relates to known microscopic parameters of EuTe4?" The authors did not address this question in their response to Comment 3, but I found in their response to referee #4 that the threshold fields were approximately 0.75 and 0.86 kV/cm, and the value is comparable to the depinning field in 2D CDW materials such as 1T-TaS2. However, it is unclear to me what is the relationship between depinning — if it occurs in EuTe4 at all — and the resistive switching observed in their experiments. I think it will benefit readers if the authors can provide some intuition about the magnitude of the threshold field even if the authors cannot nail down an exact microscopic mechanism.

Reply: We apologize for not addressing the electric field values already in a response to Comment 3. We provided the electric field values in the answer to your Comment 5, which also posed a question about the electric field.

We provided a comparison to the depinning value, as it is a widely discussed mechanism for IV nonlinearities in CDW systems and is, notably, induced in 1T-TaS2 by a value of electric field of ~ 1 kV/cm. As depinning is a volatile process with a decrease in resistance with increasing field, it can't explain the observed switching in $EuTe_4$. This is also stated in the manuscript.

In the revised manuscript, we expand the discussion regarding the threshold electric field. The electric fields needed to induce Zener tunneling are much larger (on the order of MV/cm). The observed threshold field in $EuTe_4$ is also much smaller compared to the electric field needed for reversal of out-of-plane polarization of certain van der Waals materials, such as WTe_2 where fields are on the order of MV/cm (Fei et al., Nature 560, 2018). The switching field is also lower than the avalanche breakdown field of 1-10 kV/cm observed in narrow-gap semiconductors (Guiot et al., Nat. Commun. 1722, 2013).

Concerning my Comment 8(ii)(b), which asks, "what happens microscopically when an electrical pulse passes through the sample?", the authors did not address the question. The same question is also raised by referee #1. While I understand the complexity of the exact physical scenarios involved, I think it would still be beneficial to provide some intuition about the possible scenarios or provide some constraints to exclude certain scenarios. For example, the fact that electric field polarity does not matter despite the CDW phase being polar can rule out certain scenarios where there is a direct dipolar coupling between

the electric field and the polar lattice. A more thorough discussion can benefit interested readers in joining force to pin down the exact mechanism through future works.

Reply: $EuTe_4$ offers very rich and complex physics of interacting CDWs that still needs to be worked out even in equilibrium. While the exact switching mechanism remains elusive, we have expanded the discussion by comparing our observations to currently established memristive switching mechanisms. In the previous version of the manuscript, we have already ruled out CDW sliding and Joule heating as potential switching mechanisms.

We can exclude the Zener tunnelling and avalanche breakdown as observed in dielectrics or Mott insulators, as the process is volatile, accompanied by a sub-linear IV response, and results in a lower device resistance.

Furthermore, our data show that switching is independent of polarity and there is no hysteresis in the electric field. This rules out switching due to ion migration, as observed for memristive switching in oxides, such as SrTiO3-x (Janod et al., Adv Funct Materials 25, 2015). Despite the CDW order having a polar structure, the absence of a bipolar response to the electric field also means the switching is not due to a simple in-plane polarity reversal (as observed in some ferroelectric materials).

As suggested by our theoretical analysis, the out-of-plane coupling between the CDWs is likely involved, as this naturally gives rise to distinct metastable states with different sizes of the energy gap, explaining memristive switching. However, further experiments beyond transport measurements are needed to understand the switching mechanism.

Concerning Fig. S14 in the response letter (which is labeled as Fig. S12 in the Supplementary Information), the caption says “the unit cell of the in-plane CDW phase is shown in blue.” This schematic is contradictory to the fact that the CDW is approximately tripling the unit cell only in the crystallographic b direction, so the in-plane CDW unit cell should be a rectangle that is 45-degree rotated.

Reply: We thank the reviewer for bringing our attention to this problem. We recognize that the unit cell we presented in the supplementary text is actually a supercell of the distorted $EuTe_4$ structure. We corrected our description accordingly to utilize the primitive unit cell of the distorted $EuTe_4$ structure and its corresponding momentum space counterpart, see Fig. 2 below. We did not opt for a 45-degree rotated rectangle, but an alternative choice presented in Fig. S12, as its borders are parallel to the primitive vectors (supplementary text) - Fig. 2 below.

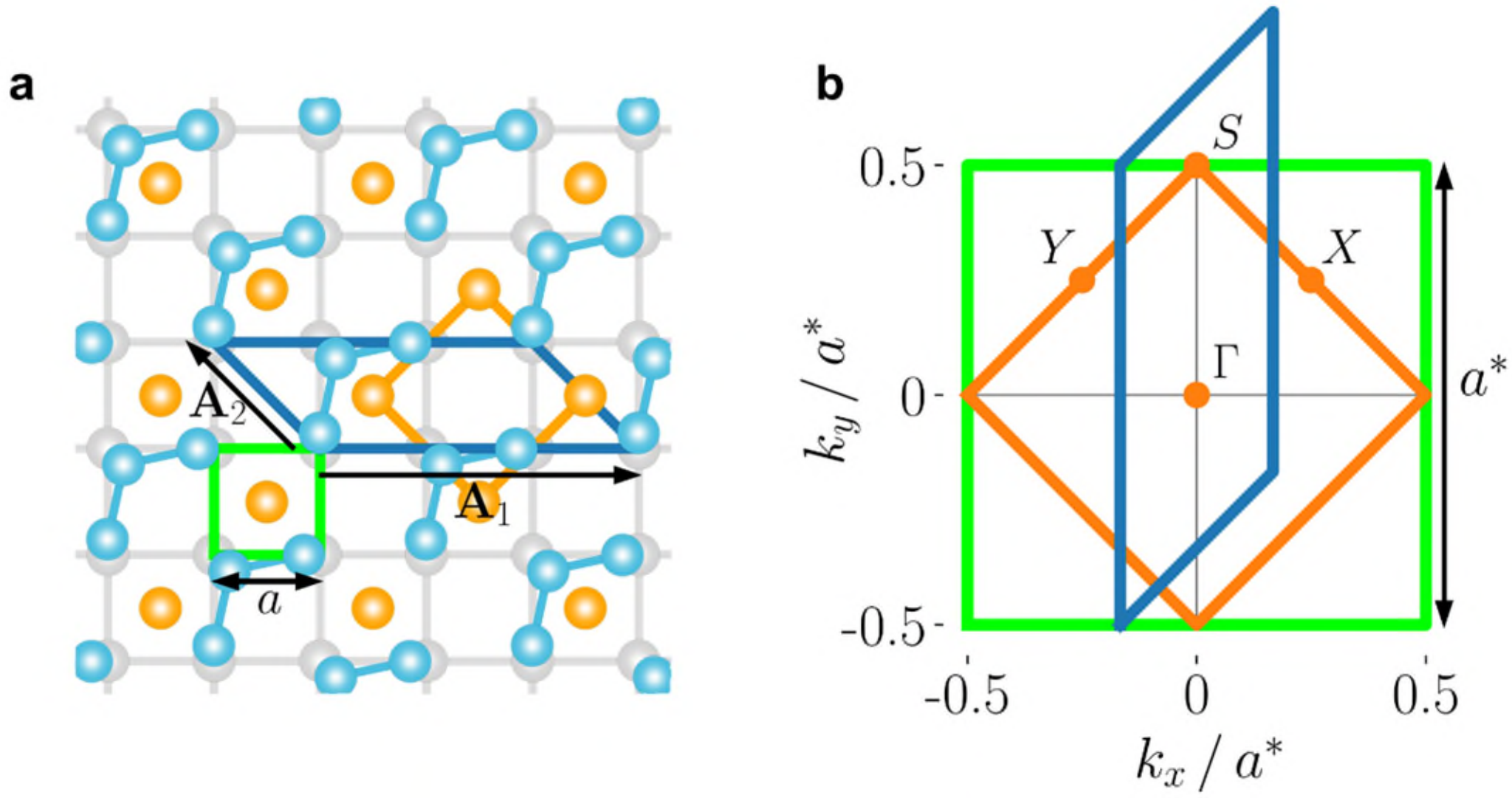

Fig. 2: The real-space (a) and momentum space (b) unit cells/Brillouin zones of the undistorted in-plane Te sheets (green), taking into account the full symmetry of the crystal (orange). The unit cell of the in-plane CDW phase for the Te sheet is shown in blue with primitive vectors marked as A_1 and A_2.

Concerning Fig. S13, how were the electronic dispersions calculated? The authors should specify the value of alpha's used in the plot. It should be noted that the schematic electronic dispersions in Fig. S13 qualitatively contradicts to the measured electronic dispersion in angle-resolved photoemission spectroscopy, see for example, Zhang et al., Phys. Rev. B 106, L201108 (2022); Lv et al., Phys. Rev. Lett. 128, 036401 (2022); Zhang et al., Phys. Rev. B 107, 115141 (2023); Elius et al. arXiv:2508.06464 (2025). The dispersions between Gamma-X and Gamma-Y are not symmetrical (see Fig. 2 in Zhang et al. 2023), and the authors should comment on how this major discrepancy impacts the tight-binding results on the electronic properties of the two CDW states.

Reply:

i) The electronic dispersions were calculated by diagonalizing the electronic Hamiltonian of a three-layer system (same as in Fig. 4b, c), with the values of the lattice distortion parameters \alpha, \theta_x, and \theta_y determined by a relaxation procedure as described in the Methods section. The values of \alpha in the electronic dispersions thus correspond to the minima presented in Fig. 4a. For completeness, we added those values to the figure caption.

ii) The perceived discrepancy between ARPES results and our calculation originates primarily from using a different unit cell in our Te-sheets-only description compared to that of the $EuTe_4$ crystal (see previous answer). This results in a different path being taken through a different reduced Brillouin zone, which thus cannot be compared directly. Motivated by the thorough response of Reviewers #4 and #5, we adapted our dispersion calculations to follow a path in the full crystal BZ, allowing for a direct comparison of our dispersions to ARPES measurements. For the convenience of the reviewers, below we provide part of Fig. 2 from Zhang et al., Phys. Rev. B 107, 115141 (2023). We note a slightly different choice of the labelling of the high-symmetry points (legend: X->Y, Y->X, M->S), and we stick with our previous labeling to avoid confusion. Below is a quick analysis of the results:

Fig. 3 Gamma-X cut equivalent to the Fig. 4 Gamma-Y cut shows one large peak near the X/Y point.

Fig. 3 Gamma-Y cut equivalent to the Fig. 4 Gamma-X cut shows one small peak near the Y/X point.

This resolves the reviewer's comment on the asymmetry of the Gamma-X and Gamma-Y dispersions, which in this (rotated) representation agrees with ARPES.

We point out that there are still the yet unresolved questions of the backfolded peak at the Gamma point, which is not seen in the cuts. Some signatures are, however, observed in Fig. 2a in Zhang et al., Phys. Rev. B 107, 115141 (2023)

Fig. 3 X-M cut equivalent to the Fig. 4 Y-S cut shows two peaks of approximately equal height, one nearer the X/Y point and the other centered at the M/S point (weaker in Fig. 3).

In total, we consider the comparison rather satisfactory, considering the simplicity of our modelling. More careful comparison would require higher resolution data at larger binding energies and a combination of our many-body approach with parameters determined from first principles, which we leave for future work.

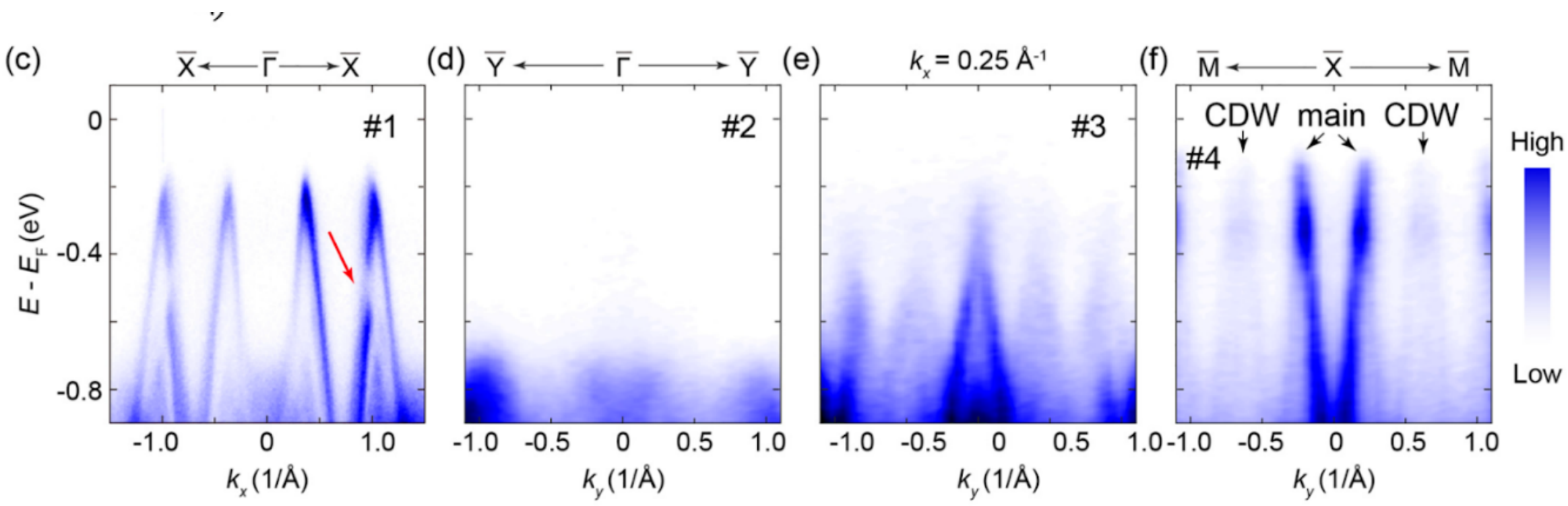

Fig. 3: ARPES measurements - part of Fig. 2 from Zhang et al., Phys. Rev. B 107, 115141 (2023). We note that the edge of the Brillouin zone in the figures occurs at k ≈ 0.7 Å-1.

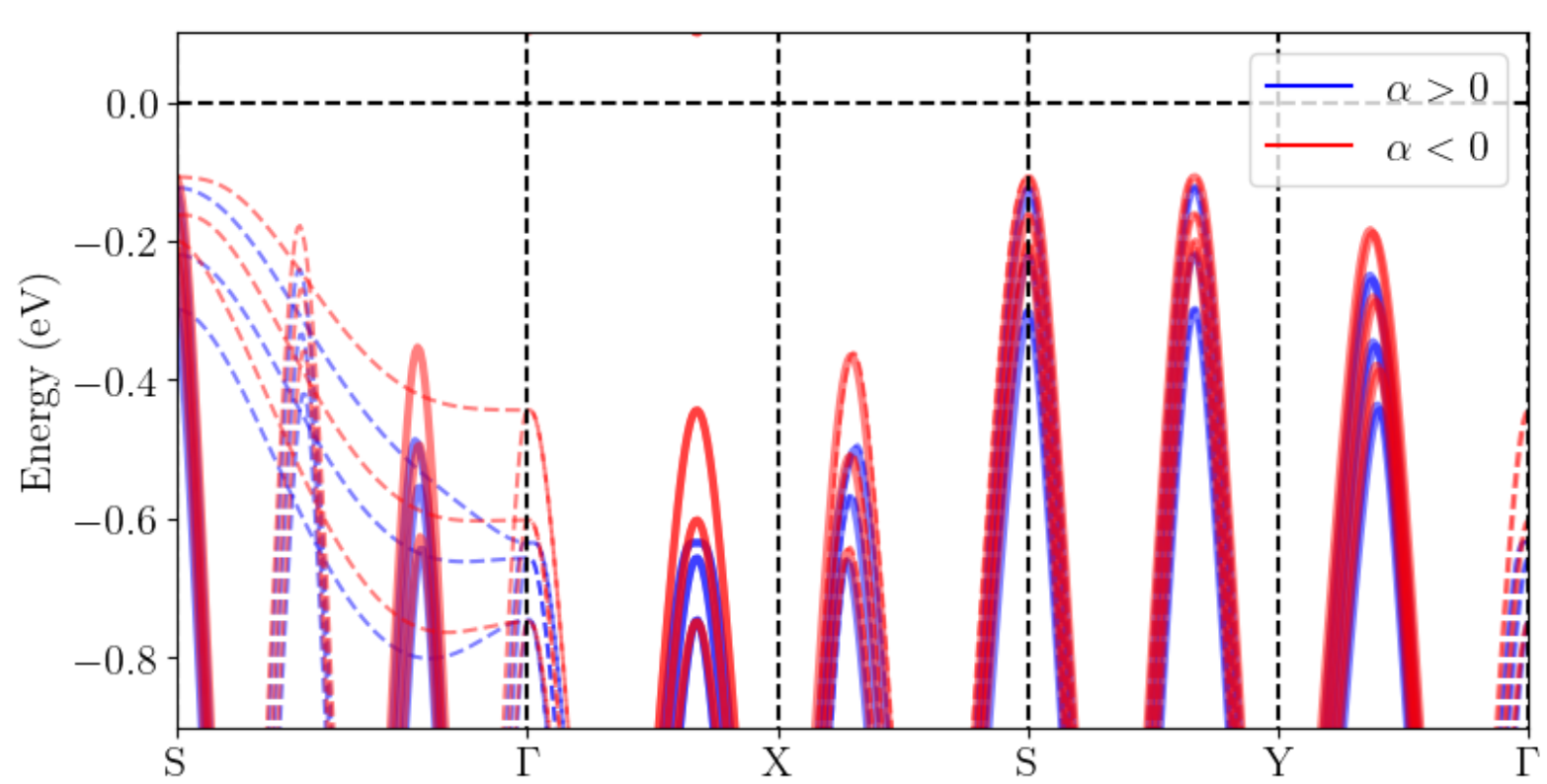

Fig. 4: Electronic dispersion of the ground state (blue, α=+0.05) and metastable state (red, α=-0.05) along a high-symmetry path in the FBZ of $EuTe_4$.

Minor comment: On page 8 of the revised manuscript, the authors wrote "i.e., rotated by 45º w.r.t. the unit cell of EuTe4 and scaled accordingly 50". The reference [50], which concerns BiFeO3 thin films, looks puzzling in this context.

Reply: We corrected the hiccup in the referencing. The correct reference the statement should be pointing at is: Brouet, V. et al. Angle-resolved photoemission study of the evolution of the band structure and charge density wave properties in RTe3 (R=Y, La, Ce, Sm, Gd, Tb and Dy), Phys. Rev. B 77, 235104 (2018).
